# Evolution of affinity between p53 transactivation domain and MDM2 across the animal kingdom demonstrates high plasticity of motif‐mediated interactions

**DOI:** 10.1002/pro.4684

**Published:** 2023-07-01

**Authors:** Filip Mihalič, Emma Åberg, Pouria Farkhondehkish, Niels Theys, Eva Andersson, Per Jemth

**Affiliations:** ^1^ Department of Medical Biochemistry and Microbiology Uppsala University Uppsala Sweden

**Keywords:** affinity, intrinsically disordered protein, MDM2, motif, p53, protein evolution, protein interaction

## Abstract

The interaction between the transcription factor p53 and the ubiquitin ligase MDM2 results in the degradation of p53 and is well‐studied in cancer biology and drug development. Available sequence data suggest that both p53 and MDM2‐family proteins are present across the animal kingdom. However, the interacting regions are missing in some animal groups, and it is not clear whether MDM2 interacts with, and regulates p53 in all species. We used phylogenetic analyses and biophysical measurements to examine the evolution of affinity between the interacting protein regions: a conserved 12‐residue intrinsically disordered binding motif in the p53 transactivation domain (TAD) and the folded SWIB domain of MDM2. The affinity varied significantly across the animal kingdom. The p53TAD/MDM2 interaction among jawed vertebrates displayed high affinity, in particular for chicken and human proteins (*K*_D_ around 0.1 μM). The affinity of the bay mussel p53TAD/MDM2 complex was lower (*K*_D_ = 15 μM) and those from a placozoan, an arthropod, and a jawless vertebrate were very low or non‐detectable (*K*_D_ > 100 μM). Binding experiments with reconstructed ancestral p53TAD/MDM2 variants suggested that a micromolar affinity interaction was present in the ancestral bilaterian animal and was later enhanced in tetrapods while lost in other linages. The different evolutionary trajectories of p53TAD/MDM2 affinity during speciation demonstrate high plasticity of motif‐mediated interactions and the potential for rapid adaptation of p53 regulation during times of change. Neutral drift in unconstrained disordered regions may underlie the plasticity and explain the observed low sequence conservation in TADs such as p53TAD.

## 1 INTRODUCTION

**FIGURE 1:**
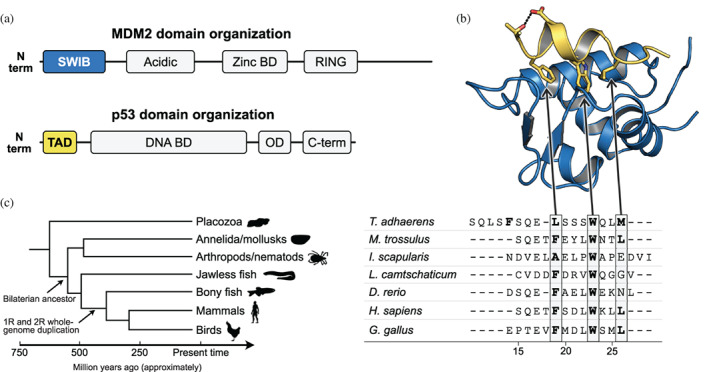
Sequences and structure of the p53TAD‐MDM2 complex. (a) Schematic of p53 and MDM2 proteins with indicated folded domains. (b) Crystal structure of a complex between human p53TAD (yellow) and MDM2 (blue). The three conserved hydrophobic residues in the p53TAD motif, Phe19, Trp23, and Leu26 as well as Thr18 and Asp21 are shown as sticks. The hydrogen bond between Thr18 and Asp21 is shown as dots (PDB code: 1YCR). (Kussie et al., 1996) (c) Schematic phylogenetic tree and sequence alignment of the most conserved region of p53TAD, which entails the canonical binding motif for MDM2, a 12‐residue intrinsically disordered region that folds into an α helix upon binding to MDM2. The motif contains three hydrophobic residues that points straight down in the binding pocket of the MDM2 SWIB domain, namely F_19_xxxW_23_xxL_26_ in human p53TAD, highlighted in gray. The four residues N‐terminal of the Phe residue, as well as Leu23 are also relatively well conserved. The numbering of motif residues is based on the human sequence throughout the paper to facilitate comparison. Note that an alternative alignment is possible for *T. adhaerens* p53TAD with a motif F_15_xxxL_19_xxxW_23_ that has one extra residue between Leu19 and Trp23. 1R and 2R represent two whole genome duplications in the vertebrate lineage. These genome duplications gave rise to the paralogs p53, p63, and p73 as well as MDM2 and MDM4 in extant vertebrates. The duplications might have occurred earlier than indicated, prior to or during (Putnam et al., 2008; Smith et al., 2013) the divergence of jawless fishes, but present‐day p53 paralogs in jawless fish appear not to correspond to those in other vertebrates. Animal silhouettes are from PhyloPic.

The transcription factor p53 is involved in many cellular processes including cell cycle regulation and apoptosis (Vogelstein et al., 2000). Because of this, mutations leading to p53 malfunction are highly correlated with cancer (Joerger & Fersht, 2016). The central position in cell cycle regulation is consistent with an ancient origin of p53, as evident from the presence of genes encoding p53‐like proteins across the animal kingdom (Joerger et al., 2014; Lane et al., 2010; Zhang et al., 2022). It has been shown in well‐studied vertebrate animals that p53 is kept at appropriate levels in healthy cells through ubiquitination and subsequent degradation (Brooks & Gu, 2006). The ubiquitination process is initiated by binding the intrinsically disordered p53 transactivation domain (TAD) to the SWIB domain of the ubiquitin ligase MDM2. The interaction is mediated by a conserved ~12‐residue motif within p53TAD that folds into an α‐helix upon binding to MDM2 (Figure 1a,b).

We have previously constructed phylogenetic trees of both the p53 and MDM2 protein families and examined their co‐evolution across the animal kingdom with emphasis on their interaction domains p53TAD and SWIB (Åberg et al., 2017). In agreement with earlier studies (Lane et al., 2010; Tan et al., 2017) we found that the interaction domains are present in distantly related animal phyla, suggesting that the interaction between p53TAD and MDM2 dates back to the beginning of animal life. Furthermore, our analysis suggested that p53TAD and the SWIB domain of MDM2 is present in all extant deuterostome animals, but that both protein regions have disappeared in distinct protostome lineages, although splice variants (Ingaramo et al., 2018) as well as coverage and quality of sequence databases complicate such analysis. In addition, while a SWIB domain‐containing protein is present in *Drosophila*, it appears not to be part of an MDM2 homolog (Lane & Verma, 2012). Among the animals that diverged before the protostome/deuterostome split, the interaction domains were not found in cnidarians and porifera. However, they are present in the placozoan *Trichoplax adhaerens*, from an evolutionary branch that diverged before the radiation of all other extant animal phyla (Figure 1c) (Lane et al., 2010). Furthermore, pull‐down and co‐transfection experiments suggested a functional p53/MDM2 interaction in *T. adhaerens*, (Siau et al., 2016) in the protostome *Mytilus trossulus* (a mollusk) (Muttray et al., 2010) and also in the non‐jawed vertebrate Japanese lamprey (Coffill et al., 2016). Thus, available data suggest an intriguing scenario where MDM2‐dependent regulation of p53TAD was present in the last common ancestor of all animals and has been maintained in most extant animal phyla, but lost in some of them.

Prior to the radiation of extant vertebrate classes around 450 million years ago (Mya) two whole genome duplication events further shaped the evolution of the p53/MDM2 interaction. The genome duplications clearly occurred before the split of cartilaginous and bony fish, (Dehal & Boore, 2005; Vandepoele et al., 2004) but possibly even earlier, before or concurrent with the divergence of jawless fish (Putnam et al., 2008; Smith et al., 2013). In either case, the present‐day paralogs p53, p63, and p73 as well as MDM2 and MDM4 likely originated in these genome duplications. The TAD in p53, p63, and p73 and the SWIB domain in MDM2 and MDM4 have been retained in extant vertebrate lineages, but evolution has subjected the proteins to sub‐ and neofunctionalization. Indeed, inspection of the amino acid sequences for vertebrate p53TAD orthologs reveals dramatic changes between different animal groups although a 12‐residue binding motif is relatively well conserved (Figures 1c and S1). Likewise, MDM2 and MDM4 display significant divergence with around 55% amino acid identity for residues within the folded part of the SWIB domain, as defined by the crystal structure of human p53TAD/SWIB (Kussie et al., 1996). A similar low identity is seen between human and lamprey MDM2 (53%), consistent with a split of jawed (gnathostomes) and non‐jawed vertebrates (agnatha, including extant lampreys) around the time of the whole genome duplications when the genes encoding MDM2 and MDM4 diverged (Kuraku et al., 2008; McLysaght et al., 2002) (Figure S1). In this paper we conform to the common naming of “p53” and “MDM2” also for non‐vertebrate homologs, although it is not formally correct. In fact, the ancestral p53/p63/p73 protein was likely more similar to extant p63 and p73 than to p53 (Zhang et al., 2022).

Conservation of sequence implies function. However, while phylogenetic methods are very powerful, the affinity of protein interactions depends on fine molecular details and needs to be investigated by experiments. Therefore, since some animals apparently have lost their p53/MDM2 interaction while those that retained it display a significant divergence in amino acid sequence in the p53TAD interaction domains, we set out to directly assess the evolution of affinity between the conserved binding motif in p53TAD and the SWIB domain of MDM2 in the animal kingdom. Using biophysical measurements on extant as well as resurrected ancient proteins we found that the amino acid changes observed along different evolutionary trajectories have indeed modulated the affinity of the interaction. The history of the p53TAD/MDM2 interaction demonstrates the evolutionary plasticity and malleability of intrinsically disordered protein regions involved in protein–protein interactions.

## 2 RESULTS

### 2.1 p53TAD/MDM2 affinity across extant animals

Sequence‐based predictions suggest that p53 and MDM2 were present in the last common ancestor of all animals (Lane et al., 2010). However, the interacting regions, p53TAD, and the MDM2 SWIB domain could not be found in sequences from insects, crustaceans, and cnidarians (Åberg et al., 2017) or only in minor splice variants (Bourdon et al., 2005) suggesting that p53 is not regulated by MDM2 in these species. To further investigate the co‐evolution between p53 and MDM2 we here mapped the interaction between p53 and MDM2 across the animal kingdom. Specifically, we measured affinity between the conserved 12‐residue binding motif in p53TAD and the SWIB domain of MDM2 from extant animals representing different lineages that retained both interaction domains: mammals (human, *Homo sapiens*), birds (chicken, *Gallus gallus*), bony fishes (Zebra fish, *Danio rerio*), jawless fishes (Arctic lamprey, *Lethenteron camtschaticum*), arthropods (Deer tick, *Ixodes scapularis*), mollusks (Bay mussel, *Mytilus trossulus*), and the multicellular placozoan *T. adhaerens*. To facilitate comparison of residues at corresponding positions we use the numbering of human p53 residues based on a sequence alignment of the 12‐residue binding motif (Figure 1c). Sequences of all constructs used in the paper are compiled in Excel File 1 in Data S1.

We used kinetics (stopped‐flow spectrometry) or equilibrium experiments (fluorescence polarization, FP, or isothermal titration calorimetry, ITC) to determine the affinity of interactions between p53TAD and MDM2.

**FIGURE 2:**
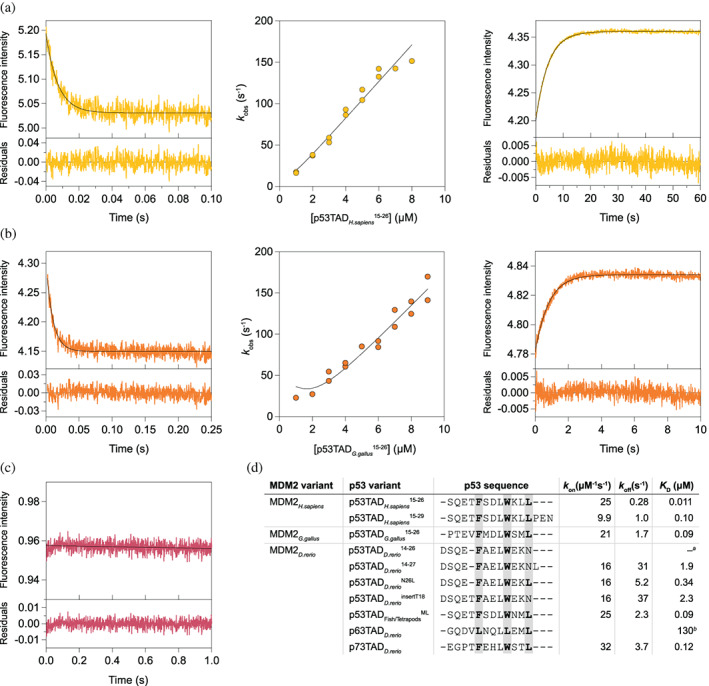
Affinity between p53TAD and MDM2 from extant vertebrate species. Binding was measured using stopped‐flow spectroscopy. (a) Representative traces for the binding (left panel) and the displacement experiments (right panel) of MDM2_*H. sapiens*_ interacting with p53TAD_*H. sapiens*_^15–26^. *k*_on_ is determined from the fitting of *k*_obs_ values obtained by performing association experiments at different peptide concentrations (middle panel). (b) Same experiments as in (a) for the chicken MDM2/p53TAD pair. (c) We could not observe any change in fluorescence upon mixing p53TAD_*D. rerio*_^14–26^ with MDM2_*D. rerio*_, indicating absence of interaction. (d) Sequence alignment and kinetic parameters for vertebrate MDM2‐p53TAD interactions. The errors are reported in Table 1. ^a^No binding could be detected with stopped‐flow spectroscopy. ^b^The affinity of p63TAD_*D. rerio*_ was measured using fluorescence polarization.

The affinity between p53TAD_*H. sapiens*_ and MDM2_*H. sapiens*_ was similar to that from previous studies (Figure 2a, Table 1). Furthermore, human and chicken p53TAD/MDM2 complexes displayed similar affinity (80–100 nM) (Figure 2b,d). In fact, p53TAD_H. sapiens_^15–26^ displays an apparently higher affinity (~10 nM) with MDM2_*H. sapiens*_, but this is an artifact resulting from the short length of the peptide. Two longer variants, p53TAD_H. sapiens_^15–29^ and p53TAD_H. sapiens_^13–61^ have *K*_D_ values of 60–100 nM (Åberg et al., 2018). This discrepancy has been previously reported by other groups (Schon et al., 2002) and suggested to be an effect of a non‐native closing of the N‐terminal lid in MDM2 over the binding cleft which is only possible with the shorter p53TAD peptide (Showalter et al., 2008). Thus, while ~100 nM is a good estimate of *K*_D_ for the native interaction between human p53TAD and MDM2, we use the short p53TAD_H. sapiens_^15–26^ (*K*_D_ ~ 10 nM) for a direct comparison of binding to MDM2s from different species (Table 2). Note that several p53TADs display this “non‐native” high affinity with MDM2_*H. sapiens*_.

**TABLE 1:** Affinity and rate constants for resurrected and extant p53TAD‐MDM2 interactions.

MDM2 variant	p53TAD variant	*K*_D_ (μM)	*k*_on_ (μM^−1^ s^−1^)	*k*_off_ (s^−1^)
**MDM2**_***H. sapiens***_	**p53TAD**_***H. sapiens***_^15–26^	0.011 ± 0.002^a^	25 ± 1	0.28 ± 0.03
**MDM2**_***H. sapiens***_	**p53TAD**_***H. sapiens***_^15–29^	0.10 ± 0.002^b^	9.9 ± 0.1	1.0 ± 0.02
**MDM2**_***G. gallus***_	**p53TAD**_***G. gallus***_^14–26^	0.09 ± 0.01	21 ± 1	1.7 ± 0.1
**MDM2**_***D. rerio***_	**p53TAD**_***D. rerio***_^14–26^	‐^c^		
	**p53TAD**_***D. rerio***_^14–27^	1.9 ± 0.1	15.9 ± 0.6	31 ± 1^d^
	p53TAD_*D. rerio*_^N26L^	0.34 ± 0.01	15.5 ± 0.3	5.2 ± 0.1
	p53TAD_*D. rerio*_^insertT18^	2.3 ± 0.4	16.1 ± 0.6	37 ± 5^e^
	p53TAD_Fishes/Tetrapods_^ML^	0.093 ± 0.01	24.6 ± 0.8	2.3 ± 0.2
	p73TAD_*D. rerio*_	0.12 ± 0.01	32 ± 1	3.7 ± 0.06
	p63TAD_*D. rerio*_	130 ± 2^f^		
**MDM2**_***L. camtschaticum***_	**p53TAD**_***L. camtschaticum***_	250 ± 32^f^		
**MDM2**_***M. trossulus***_	**p53TAD**_***M. trossulus***_	15 ± 3^f^		
**MDM2**_***I. scapularis***_	**p53TAD**_***I. scapularis***_	‐^g^		
**MDM2**_***T. adhaerens***_	**p53TAD**_***T. adhaerens***_	‐^g^		
**MDM2**_**Fishes/Tetrapods**_^**ML**^	**p53TAD**_**Fishes/Tetrapods**_^**ML**^	0.145 ± 0.007	28 ± 1	4.06 ± 0.03
MDM2_Fishes/Tetrapods_^AltAll^	p53TAD_Fishes/Tetrapods_^AltAll^	0.098 ± 0.005	30 ± 1	2.93 ± 0.07
**MDM2**_**Reptiles/Mammals**_^**ML**^	**p53TAD**_**Reptiles/Mammals**_^**ML**^	0.120 ± 0.007	25.4 ± 0.9	3.1 ± 0.1
MDM2_Reptiles/Mammals_^AltAll^	p53TAD_Reptiles/Mammals_^AltAll^	0.20 ± 0.01	18.6 ± 0.6	3.7 ± 0.2
MDM2_Fishes/Tetrapods_^ML^	p53TAD_1R_^ML^	0.66 ± 0.08	14.3 ± 0.4	9 ± 1
	p53TAD_1R_^AltAll^	0.37 ± 0.02	18.3 ± 0.6	6.8 ± 0.2
	p53TAD_Bilateria_^ML^	2.1 ± 0.4	8.7 ± 0.6	18 ± 3
	p53TAD_Bilateria_^AltAll^	0.65 ± 0.03	11.6 ± 0.3	7.5 ± 0.2

*Note*: The maximum likelihood (ML) contemporary (phylogenetic tree‐matched) and extant interactions are highlighted in bold. AltAll represents alternative variants with all uncertain positions (posterior probability <0.9) replaced with the second most likely amino acid residue. Point mutations are indicated as superscripts. Extant species in the table: zebra fish, *Danio rerio*; chicken, *Gallus gallus*; human, *Homo sapiens*; arctic lamprey, *Lethenteron camtschaticum*; bay mussel, *Mytilus trossulus*; deer tick, *Ixodes scapularis*; and the placozoa *Trichoplax adhaerens*. The p53TAD peptide corresponding to the conserved binding motif is 12 residues long and corresponds to human residues 15–26 unless otherwise specified.

^a^ The affinity for 12‐mer p53TAD_Human_ is incorrectly high, likely due to a non‐native interaction.

^b^ Data acquired at *I* = 0.20 M from Åberg et al.'s (Åberg et al., 2018).

^c^ The affinity was too low to be measured by stopped flow spectroscopy.

^d^ *k*_off_ from extrapolation = 28 ± 4 s^−1^.

^e^ *k*_off_ from extrapolation = 32 ± 3 s^−1^.

^f^ *K*_D_ from fluorescence polarization displacement experiment with low constant FITC‐labeled p53TAD_Human_, which was displaced by excess p53TAD from the respective species.

^g^ Increase in fluorescence polarization signal was observed with FITC‐labeled p53TAD_Human_^15–26^ (see Table 2), but the signal could not be reversed by unlabeled native p53TAD peptide suggesting non‐specific binding of p53TAD_Human_^15–26^. See Materials section for description of the errors.

**TABLE 2:** Affinity and rate constants for binding of human MDM2 to extant and ancient p53TAD and for binding of human p53TAD to extant and ancient MDM2 variants

MDM2 variant	p53TAD variant	*K*_D_ (μM)	*k*_on_ (μM^−1^ s^−1^)	*k*_off_ (s^−1^)
MDM2_*H. sapiens*_	p53TAD_*H. sapiens*_^15–26^	0.011 ± 0.002	24 ± 1	0.28 ± 0.03
MDM2_*H. sapiens*_	p53TAD_Bilateria_^ML^	0.6 ± 0.1	19 ± 1	11 ± 2
MDM2_*H. sapiens*_	p53TAD_Bilateria_^AltAll^	0.17 ± 0.02	15 ± 1	2.6 ± 0.3
MDM2_*H. sapiens*_	p53TAD_1R_^ML^	0.082 ± 0.006	16.7 ± 0.9	1.37 ± 0.06
MDM2_*H. sapiens*_	p53TAD_1R_^AltAll^	0.024 ± 0.002	29 ± 2	0.70 ± 0.04
MDM2_*H. sapiens*_	p53TAD_Fishes/Tetrapods_^ML^	0.008 ± 0.001	43 ± 1	0.36 ± 0.05
MDM2_*H. sapiens*_	p53TAD_Fishes/Tetrapods_^AltAll^	0.019 ± 0.001	27.3 ± 0.7	0.52 ± 0.04
MDM2_*H. sapiens*_	p53TAD_Reptiles/Mammals_^ML^	0.008 ± 0.001	42 ± 2	0.32 ± 0.04
MDM2_*H. sapiens*_	p53TAD_Reptiles/Mammals_^AltAll^	0.0095 ± 0.0005	38 ± 2	0.36 ± 0.01
MDM2_*H. sapiens*_	p53TAD_*D. rerio*_^14–26^	‐^a^		
MDM2_*H. sapiens*_	p53TAD_*D. rerio*_^14–27^	5.1 ± 0.7	4.0 ± 0.6	20.5 ± 0.1^b^
MDM2_*H. sapiens*_	p53TAD_*D. rerio*_^N26L^	0.19 ± 0.02	7.4 ± 0.7	1.4 ± 0.03
MDM2_*H. sapiens*_	p53TAD_*D. rerio*_^insertT18^	4.9 ± 0.9	6 ± 1	29 ± 1^c^
MDM2_*H. sapiens*_	p53TAD_*G. gallus*_^15–26^	0.012 ± 0.001	30 ± 2	0.32 ± 0.014
MDM2_*H. sapiens*_	p53TAD_*L. camtschaticum*_	120 ± 1^d^ ^,^ ^e^	‐	‐
MDM2_*H. sapiens*_	p53TAD_*M. trossulus*_	0.021 ± 0.002	23 ± 2	0.49 ± 0.02
MDM2_*H. sapiens*_	p53TAD_*I. scapularis*_	400 ± 130^d^ ^,^ ^e^	‐	‐
MDM2_*H. sapiens*_	p53TAD_*T. adhaerens*_	94 ± 1^d^ ^,^ ^e^		
MDM2_Fishes/Tetrapods_^ML^	p53TAD_*H. sapiens*_	0.180 ± 0.006	18.0 ± 0.6	3.24 ± 0.05
MDM2_Fishes/Tetrapods_^AltAll^		0.083 ± 0.003	22.7 ± 0.5	1.87 ± 0.06
MDM2_Reptiles/Mammals_^ML^		0.15 ± 0.01	15.7 ± 0.4	2.4 ± 0.2
MDM2_Reptiles/Mammals_^AltAll^		0.16 ± 0.02	15.4 ± 0.4	2.4 ± 0.3
MDM2_*D. rerio*_		0.086 ± 0.007	29 ± 2	2.5 ± 0.1
MDM2_*G. gallus*_		0.077 ± 0.008	14.2 ± 0.8	1.1 ± 0.1
MDM2_*H. sapiens*_		0.018 ± 0.006^e^		
MDM2_*L. camtschaticum*_		2.7 ± 0.3^f^		
MDM2_*M. trossulus*_		34 ± 1^f^		
MDM2_*I. scapularis*_		71 ± 4^f^ ^,^ ^g^		
MDM2_*T. adhaerens*_		360 ± 33^f^ ^,^ ^g^		

*Note*: The p53TAD peptide corresponding to the conserved binding motif is 12 residues long and corresponds to human residues 15–26 unless otherwise specified. The non‐native interaction leading to high affinity between p53TAD_Human_^15–26^ and MDM2_Human_ is likely present also for the other 12‐mer peptides such that relative affinities can be compared between the 12‐mer peptides corresponding to human numbering 15–26.

^a^ The affinity was too low to be measured by stopped flow.

^b^ *k*_off_ from extrapolation = 24.5 ± 3.3 s^−1^.

^c^ *k*_off_ from extrapolation = 27 ± 4 s^−1^.

^d^ The affinity was too low to be measured by stopped flow spectroscopy.

^e^ *K*_D_ from fluorescence polarization displacement experiment with low constant FITC‐labeled p53TAD_Human_, which was displaced by excess p53TAD from the respective species.

^f^ *K*_D_ from fluorescence polarization experiment with constant FITC‐labeled p53TAD_Human_ and excess MDM2 concentration.

^g^ Uncertain data since the MDM2/FITC‐labeled p53TAD_Human_ complex could not be dissociated with native p53TAD. See Materials section for description of the errors.

Human and chicken p53TAD share the same hydrophobic triad F_19_xxxW_23_xxL_26_ (human p53 numbering) within the 12‐residue binding motif, and their p53TAD/MDM2 complexes had a similar affinity. However, there were significant differences in the affinity of p53TAD/MDM2 from other animals. p53TAD_*D. rerio*_^14–26^ a 12‐mer peptide corresponding to the conserved binding‐motif region but with a deletion of Thr (or Ser) at position 18, showed non‐measurable affinity for MDM2_*D. rerio*_ with stopped‐flow spectroscopy. There is an apparent insertion of an Asn residue at position 26 in the conserved motif. Either changing Asn26 to Leu (p53TAD_*D. rerio*_^N26L^, *K*_D_ = 0.34 μM) or extending the peptide to a 13‐mer to include Leu27 (p53TAD_*D. rerio*_^14–27^ *K*_D_ = 1.9 μM) resulted in detectable binding. Lack of a Thr18 helix N‐cap also contributes to a lower affinity of the p53TAD_*D. rerio*_/MDM2_*D. rerio*_ complex as shown by a peptide where Thr18 was introduced (p53TAD_*D. rerio*_^insertT18^, *K*_D_ = 2.3 μM) (Figure 2, Table 1).

**FIGURE 3:**
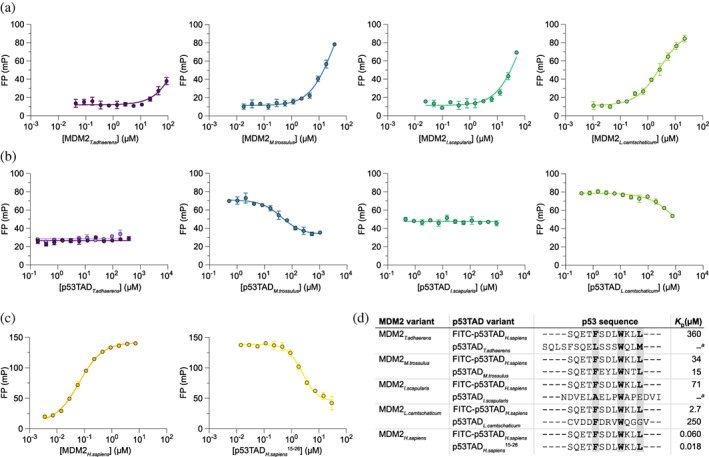
Affinity between p53TAD and MDM2 from non‐vertebrate extant species. (a) Binding between MDM2s and FITC labeled p53TAD_*H. sapiens*_^15–26^ was measured using a fluorescence polarization‐based saturation experiment. *K*_D_ for FITC labeled p53TAD_*H. sapiens*_^15–26^ was estimated from the curve fitting. (b) In the next step, FITC labeled p53TAD_*H. sapiens*_^15–26^ was competed out by an unlabeled peptide corresponding to the native p53TAD binding motif, as indicated on the x‐axis. IC_50_ values from the resulting displacement data were used to estimate *K*_D_ of the native p53TAD/MDM2 interaction. For *T. adhaerens* displacement experiments, both a short p53TAD^15–26^ (dark purple) and a full‐length p53TAD (light purple, see Excel File 1 in Data S1) were tested, but both failed to displace the weakly bound labeled peptide. (c) Saturation and displacement experiments showing the interaction between human MDM2 and p53TAD^15–26^ peptide for comparison. (d) Alignment of the binding motif of p53TAD from human, different non‐vertebrate animal species, and the jawless vertebrate arctic lamprey (*L. camtschaticum*). Approximate *K*_D_ values were determined from the data in panels a–c. Error bars for data points indicate standard deviation from technical replicates; *n* = 3. Errors for the parameters in panel d are shown in the Excel File 2 in Data S1. ^a^No binding was detected.

p53TAD/MDM2 complexes from protostomes and the non‐jawed vertebrate *L. camtschaticum* (arctic lamprey) displayed much lower affinities than the bony vertebrates. For MDM2 from *M. trossulus* (bay mussel) and *L. camtschaticum* we could observe binding with a FITC‐labeled human p53TAD^15–26^ peptide in FP experiments (Figure 3a, Table 1). This labeled peptide could then be displaced by the respective unlabeled native p53TAD_*M. trossulus*_ (*K*_D_ = 15 μM) or p53TAD_*L. camtschaticum*_ (*K*_D_ = 250 μM), yielding estimates of the native *K*_D_ values (Figure 3b). However, we could not confirm these interactions by ITC (Figure S2). Furthermore, we could not detect any binding between p53TAD and MDM2 from *T. adhaerens*, the most distantly related of all known animals, nor from the arthropod *I. scapularis* (deer tick), with either FP or ITC (Figure 3, Figure S2). For a specific interaction to the p53TAD‐binding groove in MDM2, the unlabeled peptide is expected to compete out the labeled one. However, the signal did not decrease upon addition of unlabeled native p53TAD in the case of *T. adhaerens* and *I. scapularis* so the interaction with FITC‐labeled p53TAD_H. sapiens_^15–26^ was therefore deemed non‐specific (Figure 3b).

A full‐length version of p53TAD_*T. adhaerens*_ (residues 1–126 of *T. adhaerens* p53) also did not show binding to MDM2_*T. adhaerens*_ (Figure 3b). We attempted expression and purification of full‐length p53TADs from *I. scapularis*, *M. trossulus* and *L. camtschaticum*, but could not obtain enough of pure protein for binding experiments. One problem with absence of binding is that it is impossible to completely rule out misfolding of the MDM2 SWIB domain. To minimize the risk for misfolding, SWIB domains were subjected to circular dichroism (CD) and binding experiments immediately after purification. CD spectra and CD‐monitored guanidinium chloride denaturation experiments suggested that all of the SWIB domains were soluble and folded (Figure S3), yet they displayed weak or no binding to the corresponding native p53TADs.

Structural prediction of the complexes using ColabFold(Mirdita et al., 2022) suggested that p53TAD_*M. trossulus*_, p53TAD_*L. camtschaticum*_ and p53TAD_*T. adhaerens*_ all form an alpha helix upon binding to its respective native MDM2 (Figure S4). p53TAD_*M. trossulus*_ and p53TAD_*H. sapiens*_ both have the same hydrophobic triad in the binding motif (FxxxWxxL) and the *M. trossulus* complex aligned well with the human complex. In agreement with the prediction, p53TAD_*M. trossulus*_ bound MDM2_*H. sapiens*_ with high affinity (Table 2). Close inspection of the MDM2_*M. trossulus*_ ColabFold prediction revealed several key amino acid differences in the binding pocket as compared with MDM2_*H. sapiens*_, and explained the low affinity of the native *M. trossulus* complex, despite the conserved canonical motif in p53TAD_*M. trossulus*_. These differences include Leu54 (Cys), Gly58 (Lys), Met62 (Gly), and Val93 (Phe) (numbering according to human MDM2 and with the *M. trossulus* residue in parenthesis; Figure S5), all of which should interfere with optimal binding of the p53TAD. On the other hand, the low affinity of the *L. camtschaticum* complex is due to changes in the binding motif of p53TAD_*L. camtschaticum*_ where only the first two hydrophobic residues align because of an extra residue between the conserved Trp and the third hydrophobic residue (FxxxWxxxV). p53TAD_*T. adhaerens*_ displays two putative hydrophobic triads in the conserved region (FxxxLxxxW and LxxxWxxM). A predicted complex between a peptide containing the first motif and MDM2_*T. adhaerens*_ aligned reasonably well with the human complex, while a peptide with the second motif was less well‐defined with different directions of the peptide in the structural models (Figure S4). Finally, p53TAD_*I. scapularis*_ did not adopt the typical alpha helical conformation seen in the other complexes. Instead, p53TAD_*I. scapularis*_ formed a more extended bound conformation where a Trp was found at the position of human Phe19, the conserved Leu aligned with human Trp23 and an Ala occupied the position corresponding to Leu26 resulting in a putative WxLxA motif with a reversed direction of binding compared to other p53TAD peptides (Figure S4C). Importantly, the predictions generated for the *I. scapularis* and *T. adhaerens* complexes exhibited a markedly lower pLDDT confidence score, as defined by the Colabfold algorithm, with prediction certainty parameters of 50–60 compared to >90 for other complexes (Figure S4). This low score suggested non‐conservation of the binding motif consistent with the observed lack of affinity in the binding experiments.

### 2.2 Phylogenetic reconstruction of ancient p53TAD and SWIB domain sequences

The large differences in affinity observed for the p53TAD/MDM2 interaction across the animal kingdom prompted us to investigate the evolution of affinity in greater detail in deuterostome lineages, where high affinity is observed (*K*_D_ = 100 nM–2 μM). The abundance of vertebrate sequences in public databases allowed for phylogenetic reconstruction of ancient sequences, which could then be expressed in *Escherichia coli*, purified and subjected to binding studies. Thus, to map the evolutionary trajectory of binding affinity between p53TAD and MDM2 we reconstructed and resurrected ancient contemporary (phylogenetic tree‐matched) sequences in the jawed vertebrate lineage. Maximum likelihood (ML) ancestral protein sequences were predicted for the most recent common ancestor of different extant groups of organisms based on an alignment of proteins from extant species and their relationship in a phylogenetic tree. The amino acid positions with low probability in the reconstructed sequences (Excel File 3 and 4 in Data S1) were analyzed with regard to their potential impact on binding using alternative variants denoted AltAll, (Eick et al., 2016) in which all positions with a probability below a chosen threshold (0.90) were changed to the second most likely amino acid residue (if the probability was at least 0.10). The binding properties of this “unlikely but not impossible” variant were compared to those of the corresponding ML variant to assess whether the results were robust with regard to the uncertainty in the reconstruction.

We previously generated alignments and phylogenetic trees for both p53 and MDM2 family proteins (Åberg et al., 2017) that were used in the current reconstruction. Following the whole genome duplications ≥450 Mya the vertebrate paralogs of MDM2 and MDM4 have obtained a substantial number of amino acid substitutions relative to each other resulting in a low sequence identity, which precluded reconstruction of the ancestral MDM2/MDM4 protein at 1R (Figure S1). Thus, for MDM2, we reconstructed the sequence of the common ancestor of “reptiles” (including birds, turtles, crocodiles, snakes, and lizards) and mammals living ~325 Mya, and for that of ray‐finned fishes and tetrapods (~420 Mya) (Excel File 3 in Data S1).

Likewise, the p53TAD region has experienced substantial changes in terms of substitutions, insertions, and deletions, even in evolutionarily recent times. In fact, the large number of changes precludes a reliable sequence alignment outside of the 12‐residue region that facilitates binding to MDM2 SWIB (Figures 1, S1, S6, S7, and Excel File 4 in Data S1). Thus, the 12‐residue binding motif was reconstructed for the fishes/tetrapods and reptiles/mammals nodes, allowing a direct assessment of binding to phylogenetic tree‐matched MDM2. We also reconstructed p53TAD_1R_^ML^ and p53TAD_2R_^ML^ from the time of the whole genome duplications 1R and 2R (~450 Mya), corresponding to the ancestral p53/p63/p73 protein (1R) and p63/p73 (2R), respectively (and assuming that the duplications occurred in an ancestral gnathostome). Furthermore, we reconstructed the even older p53TAD_Bilateria_^ML^ from the common ancestor of all bilaterian animals (protostomes and deuterostomes) living ~600 Mya.

### 2.3 Evolution of the p53TAD/MDM2 affinity in the vertebrate lineage

**FIGURE 4:**
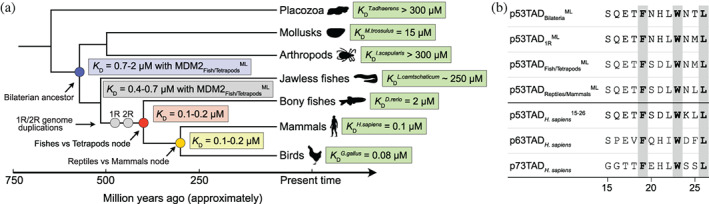
Evolution of MDM2‐p53 interaction. (a) Simplified phylogenetic tree depicting the evolution of p53TAD/MDM2 affinity. *K*_D_ values for ancestral and extant complexes in selected lineages were determined by stopped‐flow spectroscopy and fluorescence polarization experiments (see Table 1 for errors). The p53TAD in the 1R node corresponds to the most recent common ancestral p53/p63/p73 protein reconstructed from extant jawed vertebrates (gnathostomes). The genome duplications giving rise to the p53 family paralogs may have occurred prior to the split with jawless fish (agnatha), but this putative earlier p53TAD_1R_ motif could not be reconstructed due to few agnatha sequences and unclear phylogeny of the *L. camtschaticum* paralogs. (b) Alignment of the reconstructed p53TAD binding motifs and the human p53TAD, p63TAD, and p73TAD paralogs.

Reconstructed ancestral protein sequences were resurrected through peptide synthesis or expression in *E. coli*. Peptides corresponding to p53TAD^ML^ and purified MDM2^ML^s and their AltAll versions were subjected to stopped‐flow experiments in order to determine affinities. (Figure 4a, Table 1). Finally, as a common reference, we measured affinities for ancestral p53TAD variants against MDM2_*H. sapiens*_ and ancestral MDM2s against p53TAD_*H. sapiens*_^15–26^ (Table 2).

The binding affinity of the most ancient contemporary complex we could resurrect, that between MDM2_Fishes/Tetrapods_^ML^ and p53TAD_Fishes/Tetrapods_^ML^, was 145 nM (98 nM, for the AltAll complex) (Table 1). For MDM2_Reptiles/Mammals_^ML^ and p53TAD_Reptiles/Mammals_^ML^, we obtained a *K*_D_ of 120 nM (200 nM for the AltAll complex), which is very similar to that of the older fishes/tetrapod complex. Further diversification in the reptile and mammalian lineages retained or even increased the affinity of the complex, as shown by the native interactions of human and chicken p53TAD/MDM2 (Table 1), but also by the very similar (and artificially low) *K*_D_ values between MDM2_*H. sapiens*_ and extant as well as ancient reconstructed p53TADs: p53TAD_*H. sapiens*,_^15–26^ p53TAD_*G. gallus*,_^15–26^ p53TAD_Fishes/Tetrapods_^ML^, and p53TAD_Reptiles/Mammals_^ML^ (7.5–11 nM, Table 2). Furthermore, the ancestral MDM2_Fishes/Tetrapods_ and MDM2_Reptiles/Mammals_ display a similar affinity with human p53TAD (*K*_D_ = 80–180 nM) as with their reconstructed contemporary p53TADs corroborating an overall retained affinity among tetrapods.

### 2.4 Deeper evolution of the TAD

The three paralogs in the gnathostome p53 family, p53, p63, and p73, probably originated from a gene present in an ancestral vertebrate at the time of the first whole genome duplication 1R (McLysaght et al., 2002). Generally, all three proteins have the three highly conserved hydrophobic residues F_19_xxxW_23_xxL_26_ within the 12‐residue binding motif, although fish p63TADs usually contain Leu at position 23. However, outside of the binding motif, their TADs differ substantially in amino acid sequence following 450 Mya of evolution. In particular, p53TADs differ considerably between orthologs whereas p63TAD and p73TAD, respectively, are much more conserved (Figure S7). Previous experiments demonstrated that p53TAD_*H. sapiens*_ and p73TAD_*H. sapiens*_ have high affinity for MDM2_*H. sapiens*_ in contrast to p63TAD_*H. sapiens*_, which has low affinity (*K*_D_ = 6.1 μM) despite conservation of the F_19_xxxW_23_xxL_26_ motif (Åberg et al., 2018; Schon et al., 2002; Zdzalik et al., 2010). In the present study we included the p63 and p73 paralogs from *D. rerio*. Unlike p53TAD_*D. rerio*_, the conserved motif of its paralog p73TAD_*D. rerio*_ retained high affinity toward MDM2_*D. rerio*_ (*K*_D_ = 0.12 μM). p73TAD_*D. rerio*_ has the F_19_xxxW_23_xxL_26_ motif whereas p63TAD_*D. rerio*_ has a Leu residue in all three positions and very low if any affinity for MDM2_*D. rerio*_ (*K*_D_ > 100 μM as estimated by fluorescence polarization) (Figure 2D). While there is some remaining redundancy in function, p53, p63, and p73 provide an example of gene duplication(s) followed by neofunctionalization, that is, divergent evolution resulting in new functions where p53 is more involved in cell cycle arrest and apoptosis while p63 and p73 play a role in development (Levrero et al., 2000; Rozenberg et al., 2020; Rozenberg et al., 2021).

We could not resurrect 1R and bilaterian MDM2 variants. Therefore, the affinities of the reconstructed p53TAD_1R_^ML^ and p53TAD_1R_^AltAll^ (from the most recent ancestral p53/p63/p73 protein) and of p53TAD_Bilateria_^ML^ and p53TAD_Bilateria_^AltAll^ (from the most recent ancestral bilaterian p53), were measured toward MDM2_*H. sapiens*_ and MDM2_Fishes/Tetrapods_^ML^ (Tables 1 and 2). p53TAD_1R_ showed higher affinity for both MDM2_*H. sapiens*_ (*K*_D_ = 24–82 nM) and MDM2_Fishes/Tetrapods_^ML^ (*K*_D_ = 370–660 nM) than p53TAD_Bilateria_ (*K*_D_ = 170–600 nM and 1.3–1.7 μM, respectively). Thus, in particular p53TAD_Bilateria_ binds much weaker than the historically younger p53TAD_*H. sapiens*_,^15–26^ p53TAD_*G. gallus*_,^14–26^ p53TAD_Fishes/Tetrapods_^ML^, and p53TAD_Reptiles/Mammals_^ML^. Comparison of sequences (Figure 4b, Excel File 4 in Data S1) suggests that two substitutions underlie the increased affinity of the younger p53TADs. First, Ser (or Thr) at position 25 was replaced by the hydrophobic residue Met (or possibly Gln), before the genome duplications and later with Leu in the tetrapod lineage. Second, the crystal structure of the human p53TAD/MDM2 interaction shows that Thr18 in p53TAD hydrogen bonds intramolecularly with Asp21, stabilizing the bound helix conformation (Kussie et al., 1996). The present work suggests that His21 → Asp was the original substitution that allowed formation of an N‐cap, resulting in higher affinity, and which occurred between the genome duplications and the divergence of bony fishes and tetrapods. We also note that p53TAD_2R_^ML^ (which is the most recent ancestral p63/p73 protein) was very similar to p53TAD_1R_^ML^, but with a higher probability of Asp instead of Glu at position 17 (Excel File 4 in Data S1), reflecting that the genome duplications 1R and 2R likely occurred relatively close in time, irrespective of when the agnatha lineage branched off.

## 3 DISCUSSION

The evolution of functional traits is very complex and depends on interactions between multiple proteins. Despite the complexity, deciphering the molecular evolution of proteins can inform about structure–function relationships and evolutionary mechanisms governing their emergence (Harms & Thornton, 2013; Siddiq et al., 2017; Steube et al., 2023). Intrinsically disordered regions (Wright & Dyson, 2015) in proteins often contain motifs recognized by folded interaction domains (Fuxreiter et al., 2004; Tompa et al., 2014). The interaction between disordered binding motifs and domains are common in signaling pathways and transcriptional regulation and several aspects make such protein–protein interactions involving disordered protein regions particularly interesting from an evolutionary perspective. For example, except for certain key positions, such interactions appear robust to changes in the amino acid sequence increasing the possibility for permissive neutral substitutions, which may allow new functions to evolve upon further mutation and selection (Hultqvist et al., 2017). In addition, interactions of this type are often hijacked by viral proteins, (Mihalic et al., 2023) potentially imposing additional selection pressure. While we here attempted to pinpoint historical amino acid substitutions shaping the affinity between the p53TAD binding motif and MDM2, as done for other short motifs, (Laursen et al., 2021) conformational heterogeneity among the bound conformations of p53TAD is also likely contributing to the variation in affinity. Such structural plasticity, previously observed in evolution of protein–protein interactions, (Hultqvist et al., 2017; Jemth et al., 2018) is likely connected to general frustration of binding involving disordered regions, where several possible conformations compete and where none is perfectly optimized (Gianni et al., 2021).

Our present study suggests an ongoing and dynamic evolution of affinity in the interaction between the intrinsically disordered p53TAD and the folded SWIB domain of MDM2. In particular, we observe a wide range of affinities for the p53TAD/MDM2 complex among extant bilaterian animals. This raises the question about the affinity of the ancestral bilaterian p53TAD/MDM2 complex present 600 Myr. Since reconstruction of an ancestral bilaterian MDM2 was not possible, we cannot be confident about the affinity of the ancestral complex. However, based on experimental data obtained with the reconstructed ancient p53TAD_Bilateria_^ML^, p53TAD_Bilateria_^AltAll^, and MDM2_Fishes/Tetrapods_^ML^, and with the extant MDM2_H. sapiens_ we speculate that the conserved binding motif in the bilaterian complex had a *K*_D_ value in the low μM range. Subsequently, higher affinity evolved in the lineage leading to fishes and tetrapods by formation of an N‐cap in the p53TAD helix between position 18 and 21, and new hydrophobic interactions formed by the residue at position 25. After the split of the paralogs p53, p63, and p73 in an ancestral vertebrate, further evolution occurred in the motif in the respective paralog. For example, human and *D. rerio* p73TAD have high affinity for MDM2 and can likely form the N‐cap, whereas p63TAD lacks hydrogen bond donor/acceptors at the corresponding positions, which results in a shorter helix and lower affinity (Shin et al., 2015). Moreover, the canonical binding motif in p53TAD_*D. rerio*_ has evolved a lower affinity for MDM2 by loss of the hydrogen bond donor at position 18. In addition, an Asn residue at position 26 in the conserved interaction motif of p53TAD_*D. rerio*_, instead of Leu, further reduces affinity for MDM2. Nevertheless, the regulatory interplay between p53 and MDM2 is present: Previous studies have shown that DNA damage activates p53 in *D. rerio* (Lee et al., 2008) and that knockdown of MDM2 in embryos is lethal due to p53 activation and apoptosis (Langheinrich et al., 2002; Chua et al., 2015). Turning to non‐vertebrates, the p53/MDM2 system appears functional in mollusks (Muttray et al., 2010) and in *T. adhaerens* (Siau et al., 2016) despite the very low or even absent apparent affinity between their p53TAD and MDM2 SWIB domains. The low affinity can be explained by either a degenerate binding motif in p53TAD (*L. camtschaticum*), a suboptimal binding pocket in MDM2 (*M. trossulus*) or both (*I. scapularis* and *T. adhaerens*). The question arises: How can the observed radical differences in p53TAD/MDM2 affinity across bilaterian animals be compatible with a similar function? Obviously, while pivotal for binding to occur, affinity from interactions formed in the binding interface is not the sole determinant of a protein interaction. Concentrations of the interacting proteins could be regulated to compensate for suboptimal affinity. Affinity could also be modulated by disordered regions and domains outside of the central binding interface (Bugge et al., 2020; Karlsson et al., 2022). Indeed, for p53 and MDM2, an interaction between the DNA‐binding domain of p53 and the acidic domain of MDM2 was identified (Shimizu et al., 2002) and shown to be important for ubiquitination (Wallace et al., 2006). This interaction may directly increase the affinity between the proteins and be part of the co‐evolution of all factors influencing p53 regulation. Nevertheless, for the human interaction, and focusing on the TAD, the conserved binding motif is sufficient for high‐affinity MDM2 binding with little contribution from flanking regions, including the second less‐conserved binding motif AD2, which binds CBP/p300 (Åberg et al., 2018; Schon et al., 2002; Teufel et al., 2007; Teufel et al., 2009; Ferreon et al., 2009). In addition, we observed no binding with a full‐length p53TAD from *T. adhaerens*. Thus, the lack of measurable p53TAD/MDM2 affinity in proteins from *T. adhaerens* and deer tick as well as the low affinity in Japanese lamprey and bay mussel p53TAD/MDM2 is intriguing. One possibility is that p53 is not controlled by MDM2 in all species, or at least that the p53TAD/MDM2 interaction is not necessary. The observed low affinity could then be a relic from an earlier functional interaction in the most recent bilaterian ancestor. In the insect *Drosophila melanogaster* and the nematode *Caenorhabditis elegans*, which both lack a gene encoding MDM2, p53 is regulated by other proteins (Lane & Verma, 2012; Brodsky et al., 2000; Sekelsky et al., 2000), showing that a loss of MDM2‐mediated p53 regulation is biologically feasible. It is therefore not unreasonable that the functional interaction between p53 and MDM2 has been lost in other arthropod subphyla closely related to insects, such as Chelicerata (ticks, spiders), although the interaction domains are still present in the proteins. However, as noted above, other interaction surfaces may promote the association between p53 and MDM2 and maintain the regulation.

The dynamic evolution of p53TAD affinity, as illustrated by the changes at positions 18 and 25, is also visible in its phosphorylation sites. Phosphorylation of Thr18 has been shown to reduce affinity for MDM2 while increasing affinity for the transcriptional coregulators CBP/p300 (Teufel et al., 2009; Ferreon et al., 2009). Furthermore, phosphorylation of Thr18 is contingent on prior phosphorylation of Ser15 (Sakaguchi et al., 2000). In agreement with these findings, both Ser15 and Thr18 (or Ser18) are highly conserved among vertebrates, but, surprisingly, both have been lost in the bird clade (Figure S6). Another example involving phosphorylation is an apparently recent innovation in the primate lineage at Thr55, positioned in the second interaction motif AD2, which is much less conserved than the N‐terminal motif. In human p53, AD2 was recently shown to regulate DNA binding via an intramolecular interaction with the adjacent DNA‐binding domain, thereby promoting specific over non‐specific DNA binding (Krois et al., 2018; He et al., 2019). Phosphorylation of Thr55 further modulates this interaction by increasing the affinity of AD2 for the DNA‐binding domain and thereby impeding binding cooperativity for the tetrameric p53 to DNA (Sun et al., 2021). Thr55 (or Ser55) is present in our closest relatives (monkeys, lemurs, and tarsiers). However, p53s from mouse, rat (Asp55), and dog (Glu55) have a permanent negative charge in the corresponding position, suggesting a constitutively high affinity for the DNA‐binding domain (Figure S6).

Overall, the large differences in the p53TAD sequence observed among extant animals as well as the observed differences in affinity between the binding motif and the SWIB domain of MDM2 demonstrate high evolutionary plasticity in p53TAD and in its interaction with MDM2. By inference, the interactions between p53TAD and the transcriptional coregulators CBP/p300 may also be subject to changes over evolutionary time, as suggested by the striking changes at regulatory phosphorylation sites in p53TAD. In general, transcription factors regulating development and morphological phenotype are conserved (Carroll, 2008). It is interesting to contrast the low conservation of p53TAD with the much higher conservation in p63TAD and p73TAD (Figure S7), which (in particular p63) are proposed to be involved in development (Levrero et al., 2000) and protection of DNA in the germ line, the likely ancestral function (Levine, 2020). Apparently, following the genome duplications 450 Myr, in the ancestral gnathostome lineage, p53 evolved toward a new and central role in monitoring DNA damage in somatic cells. On the face of it, the high evolutionary rate of changes in the amino acid sequence in p53TAD, evident from the comparison of different extant tetrapods (Figure S6), is hard to reconcile with its interactions with both MDM2 and CBP/p300, and the centrality of p53 as a pleiotropic hub protein involved in multiple pathways (Levine, 2020). However, recent studies have shown that the exact sequence of TADs are often not crucial for binding coactivators as long as key elements are present in the binding motif, in particular hydrophobic residues (Trp, Leu) separated by negatively charged Asp or Glu (Zarin et al., 2017; Sanborn et al., 2021; Staller et al., 2022; Már et al., 2023). It has also been shown that hub proteins such as p53 that connect different biological “modules” (intermodule hubs) are generally less constrained and evolve faster than hub proteins regulating a specific biological process (intramodule hubs). (Han et al., 2004; Fraser, 2005) Furthermore, it is likely that p53 has slightly different roles related to life cycle and lifespan even among vertebrates. Thus, considering these points, evolution of p53TAD by relative fast neutral drift in unconstrained disordered regions (Brown et al., 2010) may underlie functional adaptation in different lineages and explain the observed low sequence conservation in p53TAD and variation in affinity for MDM2.

## 4 EXPERIMENTAL PROCEDURES

### 4.1 Ancestral sequence reconstruction

Sequence alignment of p53 was conducted in Guidance using the MAFFT algorithm with advanced options where the pairwise alignment option set to local pair and max‐iterate option set to 1000 iterations. The full alignment contained 342 species (Text Files 1 and 2 in Data S1). The alignment was modified by removing gaps with a gap tolerance set to 95%. For p53, the alignment of bird sequences was manually curated such that they grouped with reptiles, which are closer relatives than mammals according to the established tree of life. Furthermore, echinoderm, hemichordate, and lamprey sequences were removed since they did not group according to the tree of life. A phylogenetic tree was generated in MEGA X using maximum likelihood and the JTT substitution model. The tree was rooted against p53 from *T. adhaerens* and used to reconstruct ancestral sequences. The MDM2 alignment was performed with ClustalO and included sequences from 123 species (Text File 3 in Data S1). A maximum likelihood tree was generated (JTT, MEGA X) and rooted against MDM2 from *Callorhinchus milli*, a chondrichthyes (cartilaginous fish). The alignment and phylogenetic tree were updated for MDM2 using additional fish and bird sequences as compared to the previous study (Åberg et al., 2017). Thus, ancestral reconstruction was performed using MEGA X to obtain maximum likelihood sequences for p53TAD and the SWIB domain of MDM2, which are presented with posterior probabilities in Data S1 (Excel File 3 and 4 in Data S1). AltAll variants of the reconstructed sequences included residues for which the posterior probability of the ML residue was lower than 0.90 and where the probability for the second most likely residue was at least 0.10.

The reconstructed sequences for p53TAD from the most recent common ancestor of fishes and tetrapods (p53TAD_Fishes/Tetrapods_^ML^) showed high confidence in many positions such as the three key residues defining the core motif: F_19_xxxW_23_xxL_26_. On the other hand, the residues at positions 20, 21, and 25 were less certain (Excel File 4 in Data S1). Two p53TADs were included in reconstructed ancient complexes (where both p53TAD and MDM2 were reconstructed): p53TAD_Fishes/Tetrapods_^ML^ and p53TAD_Reptiles/Mammals_^ML^. The second most probable residues were included in p53TAD_Fishes/Tetrapods_^AltAll^ at two positions (Ser18 and Leu25) and at one position (Met18) in p53TAD_Reptiles/Mammals_^AltAll^. The ML sequences from 1R and 2R differed at one position and since p53TAD_2R_ represents the ancestral p63/p73 motif, only p53TAD_1R_ was used in the experiments, since it represents the ancestral gnathostome p53/p63/p73 motif. Both p53TAD_1R_ and the older p53TAD_Bilateria_ were reconstructed with less confidence than the more recent p53TAD_Fishes/Tetrapods_ and p53TAD_Reptiles/Mammals_.

The reconstructed MDM2 variants used in the present study correspond to residues 17–125 of human MDM2, that is, the ones visible in the crystal structure. Out of these 109 residues, 29 residues had a posterior probability below 0.90 in the MDM2_Fishes/Tetrapods_^ML^ variant and six residues a probability below 0.50. The majority of the uncertain residues are in either the N‐ or C‐terminus. In the MDM2_Reptiles/Mammals_^ML^ variant, 12 residues had a posterior probability less than 0.90 of which only one was below 0.50 (the last one in the sequence). AltAll variants contained 23 (MDM2_Fishes/Tetrapods_^AltAll^) and 12 (MDM2_Reptiles/Mammals_^AltAll^) substitutions as compared to their respective MDM2^ML^ variant (Excel File 3 in Data S1).

### 4.2 ColabFold predictions

We used the ColabFold: AlphaFold2 using MMseqs2 (Mirdita et al., 2022; Jumper et al., 2021; Evans et al., 2021) to predict complexes between the SWIB domain of MDM2 and peptides corresponding to the conserved binding motif in p53TAD, from different species. The structures of MDM2 were similar to that of the crystal structure of the human MDM2/p53TAD complex (Kussie et al., 1996). p53TAD peptides were often, but not always predicted to bind in a similar fashion as in the human complex. The predictions were used to aid sequence alignment and interpretation of binding data as described in the Results section.

### 4.3 Protein expression and purification

Extant and reconstructed cDNA encoding MDM2 variants were purchased from GenScript in either a pSY10 plasmid resulting in a construct with an N‐terminal NusA domain followed by a TEV protease site, a His‐tag, a PreScission protease site and the MDM2 variant: NusA‐TEV‐His_6_‐PreScission‐MDM2, or a pETM33 plasmid resulting in His_6_‐GST‐PreScission‐MDM2 construct. Thus, the MDM2 SWIB domains shown in Figure 1 contains GPGS or GPMG at the N‐terminus after PreScission digestion. A complete list of constructs is provided in Excel File 1 in Data S1. The cDNA was transformed into *Escherichia coli* BL21 (DE3) pLys or *Escherichia coli* BL21 (DE3) cells (Invitrogen) using heat‐shock. The cells were grown in LB medium at 37°C and overexpression of the fusion protein was induced with 1 mM isopropyl‐β‐D‐thiogalactopyranoside when the optical density at 600 nm reached 0.7–0.8. The induced cultures were incubated overnight in a rotary shaker at 18°C. Cells were centrifuged and the pellet was resuspended in a binding buffer (400 mM sodium chloride, 50 mM sodium phosphate, pH 7.8, 10% glycerol) followed by sonication to lyse the cells. Thereafter, cells were centrifuged at 4°C to remove cell debris and the supernatant was filtered and loaded onto a Nickel Sepharose Fast Flow column (GE Healthcare) in the case of pSY10 constructs. The fusion protein was eluted using binding buffer with 250 mM imidazole and then further purified using size‐exclusion chromatography on a Hi load 16/60 Sephacryl S‐100 column (GE Healthcare) in the binding buffer with pH adjusted to 7.4. The fusion protein was then cleaved with PreScission (produced in house) protease overnight at 4°C followed by a second run on the size‐exclusion chromatography column to remove the NusA protein. in the case of pETM33 constructs, after removing cell debris the supernatant was loaded onto Pierce™ Glutathione Agarose beads (Thermo Scientific), washed with wash buffer (50 mM Tris, 300 mM NaCl, pH 7.8) and eluted in GST elution buffer (50 mM Tris, 300 mM NaCl, 10 mM reduced glutathione, pH 7.8). Fusion protein was cleaved with PreScission protease overnight at 4°C and applied to Nickel Sepharose Fast Flow column to remove the tag. To remove any residual impurities final step of size‐exclusion chromatography on a Hi load 16/60 Sephacryl S‐100 column (GE Healthcare) in the binding buffer with pH adjusted to 7.4 was employed. Purity of all samples was checked with SDS‐PAGE and MALDI‐TOF mass spectrometry and pure samples were dialyzed against the experimental buffer (20 mM sodium phosphate, pH 7.4, 150 mM NaCl, 1 mM TCEP). Protein concentration was determined by measuring the absorbance at 280 nm and using extinction coefficients calculated from the amino acid sequence.

All p53TAD peptides were ordered as acetylated peptides (Ontores Biotechnology, GL Biochem Shanghai Ltd, and GeneCust [France]) and dissolved in the experimental buffer (20 mM sodium phosphate, pH 7.4, 150 mM NaCl). The peptide identity was checked with MALDI‐TOF mass spectroscopy and the concentration was determined by measuring Trp absorbance at 280 nm.

### 4.4 Kinetic experiments

Affinity from kinetics was determined from measured values of association and dissociation rate constants (*K*_D_ = *k*_off_/*k*_on_), which can be determined with high precision and accuracy in stopped‐flow spectroscopy experiments (Åberg et al., 2018). All experiments were performed at 10°C in 20 mM sodium phosphate, pH 7.4, 150 mM NaCl with addition of 1 mM of the reducing agent TCEP (experimental buffer). Kinetic experiments were performed in order to determine the association and dissociation rate constants, *k*_on_ and *k*_off_, and the dissociation equilibrium constant, *K*_D_ (i.e., as the ratio of the rate constants, *k*_off_/*k*_on_). All experiments were performed on an upgraded SX‐17 MV stopped‐flow spectrometer (Applied Photophysics). The binding was monitored using the change in emission of Trp23 in p53TAD which binds in a hydrophobic pocket of MDM2. The excitation wavelength was set to 280 nm and emission was monitored at 330 ± 25 nm using an optical interference (band‐pass) filter. The rate constants, *k*_on_ and *k*_off_, were determined in separate experiments. In both cases, the change in fluorescence was recorded with time and fitted to a single exponential equation to extract the observed rate constant *k*_obs_. To determine the association rate constant, MDM2 was held at a constant concentration of 0.5–2 μM (depending on the affinity of the complex and the change in fluorescence upon binding) and mixed with varying p53TAD concentrations in the range 1–10 μM. The observed rate constant, *k*_obs_, was plotted against the p53TAD concentration and fitted to a reversible bimolecular interaction (Malatesta, 2005), from which *k*_on_ was extracted. To determine the dissociation rate constant *k*_off_, a pre‐formed complex of p53TAD and MDM2 (1 μM:1 μM) was mixed with excess (10–20 μM) of an N‐terminally dansylated human p53TAD peptide (*D*‐ETFSDLWKLLP), which displaced unlabeled p53TAD in the complex. The observed rate constant *k*_obs_ was plotted against the concentration of dansylated p53TAD and *k*_off_ was determined at high concentrations of displacer, where it equals *k*_obs_. For low‐affinity complexes, where *k*_off_ was >20 s^−1^, *k*_off_ could be independently determined from binding experiments and shown to be similar to those from displacement experiments. *k*_on_ values are given with the curve fitting error and errors in *k*_off_ are standard deviation from two or three replicates (displacement experiment) or fitting error (extrapolation in binding experiment). Errors for *K*_D_ values (= *k*_off_/*k*_on_) were calculated from propagating the errors of *k*_off_ and *k*_on_.

### 4.5 Fluorescence polarization experiments

FP can detect weak affinities (*K*_D_ values in the high μM range), although the accuracy is lower than for the other methods. In FP experiments we observed direct binding using a FITC‐labeled p53TAD peptide and used unlabeled peptides containing the binding motif to displace the labeled one. FP experiments were performed at room temperature using the same buffer as kinetic experiments. For saturation experiments the FITC labeled human peptide was held at a constant concentration of 6 or 10 nM and mixed with the 1:1 dilution series of different MDM2 proteins (the highest concentration of proteins were 89, 37, 50, 22, and 7.3 μM for *T. adhaerens, M. trossulus, I. scapularis, L. camtschaticum* and *H. sapiens* MDM2, respectively) in black, non‐binding surface, flat bottom 96‐well plates (Corning Life Sciences). The polarization was measured at excitation/emission wavelengths of 485/535 nm on a SpectraMax iD5 plate reader. Saturation binding curves were fitted to a hyperbolic binding equation to obtain *K*_D_. For the displacement experiments, the concentration of labeled human peptide and MDM2 protein (p53TAD_*H. sapiens*_^15–26^ peptide at 6 or 10 nM, MDM2 proteins at 50, 25, 32, 10, and 2 μM for *T. adhaerens*, *M. trossulus*, *I. scapularis*, *L. camtschaticum*, and *H. sapeins* MDM2, respectively) were held constant and mixed with the 1:1 dilution series of the competing peptides (highest concentrations of peptides were 382, 187, 1060, 924, 775, and 29.5 μM for *T. adhaerens*, *T. adhaerens* full length, *M. trossulus*, *I. scapularis*, and *L. camtschaticum* p53TAD, respectively). The resulting displacement curve was fit to a sigmoidal dose‐response (variable slope) equation to obtain an IC_50_ value, which was in turn used to calculate *K*_D_ values for the competing peptides as described previously(Nikolovska‐Coleska et al., 2004) (Excel File 2 in Data S1). Errors were calculated as standard error of mean (SEM) of calculated *K*_D_ values for the displacer peptides resulting from independent fit of IC_50_ values for the replicate measurements.

### 4.6 Isothermal titration calorimetry

ITC experiments were performed with MicroCal iTC200 (GE Healthcare) at 25°C. To minimize buffer mismatch the MDM2 proteins and p53TAD peptides were dialyzed against experimental buffer prior to experiments. The concentrations of MDM2 proteins in the cell was 59.4, 57.2, 62, and 44 μM for *T. adhaerens*, *M. trossulus*, *I. scapularis*, and *L. camtschaticum* MDM2, respectively and the concentration of p53TAD peptides in the syringe was 594, 587, 645, and 440 μM for *T. adhaerens*, *M. trossulus*, *I. scapularis*, and *L. camtschaticum* p53TAD, respectively. The data were analyzed using the built‐in software and the two‐state binding model was assumed.

### 4.7 Circular dichroism spectroscopy

To assess the secondary structure content of various MDM2 domains, CD was monitored between 200 and 250 nm with a 1 nm bandwidth, scanning speed 50 nm/min and data pitch 1 nm. Experiments were performed on a J‐1500 spectrometer (JASCO) in an experimental buffer at 25°C and at 20 μM MDM2 concentration. To monitor protein unfolding the proteins were mixed with increasing concentration of guanidinium chloride (GdnCl) up to a final concentration of 6 M and the CD signal was measured at 222 nm. The data (CD signal vs. GdnCl concentration) were analyzed according to a two‐state unfolding mechanism to obtain *m*_D‐N_ value, and (GdnCl) midpoint of denaturation (Fersht, 1999). A cooperative sigmoidal denaturation suggested that the MDM2 proteins were folded in the experimental buffer.

## AUTHOR CONTRIBUTIONS

*Conceptualization, analysis, writing*: Filip Mihalič, Emma Åberg, and Per Jemth. *Methodology*: Filip Mihalič, Emma Åberg, Pouria Farkhondehkish, Niels Theys, and Eva Andersson.

## CONFLICT OF INTEREST STATEMENT

The authors declare that they have no conflict of interest with the contents of this article.

## Supporting information


**Appendix S1:** Supporting Information

**Appendix S2:** Supporting Information

**Appendix S3:** Supporting Information

**Appendix S4:** Supporting Information

**Appendix S5:** Supporting Information

**Appendix S6:** Supporting Information

**Appendix S7:** Supporting Information

**Appendix S8:** Supporting Information

## References

[pro4684-bib-0022] Åberg E , Karlsson OA , Andersson E , Jemth P . Binding kinetics of the intrinsically disordered p53 family transactivation domains and MDM2. J Phys Chem B. 2018;122:6899–905. 29878773 10.1021/acs.jpcb.8b03876

[pro4684-bib-0007] Åberg E , Saccoccia F , Grabherr M , Ore WYJ , Jemth P , Hultqvist G . Evolution of the p53‐MDM2 pathway. BMC Evol Biol. 2017;17:177. 28774266 10.1186/s12862-017-1023-y PMC5543598

[pro4684-bib-0021] Bourdon J‐C , Fernandes K , Murray‐Zmijewski F , Liu G , Diot A , Xirodimas DP , et al. p53 isoforms can regulate p53 transcriptional activity. Genes Dev. 2005;19:2122–37. 16131611 10.1101/gad.1339905 PMC1221884

[pro4684-bib-0053] Brodsky MH , Nordstrom W , Tsang G , Kwan E , Rubin GM , Abrams JM . Drosophila p53 binds a damage response element at the reaper locus. Cell. 2000;101:103–13. 10778860 10.1016/S0092-8674(00)80627-3

[pro4684-bib-0006] Brooks CL , Gu W . p53 ubiquitination: MDM2 and beyond. Mol Cell. 2006;21:307–15. 16455486 10.1016/j.molcel.2006.01.020 PMC3737769

[pro4684-bib-0067] Brown CJ , Johnson AK , Daughdrill GW . Comparing models of evolution for ordered and disordered proteins. Mol Biol Evol. 2010;27:609–21. 19923193 10.1093/molbev/msp277 PMC2822292

[pro4684-bib-0046] Bugge K , Brakti I , Fernandes CB , Dreier JE , Lundsgaard JE , Olsen JG , et al. Interactions by disorder – a matter of context. Front Mol Biosci. 2020;7:110. 32613009 10.3389/fmolb.2020.00110 PMC7308724

[pro4684-bib-0059] Carroll SB . Evo‐devo and an expanding evolutionary synthesis: a genetic theory of morphological evolution. Cell. 2008;134:25–36. 18614008 10.1016/j.cell.2008.06.030

[pro4684-bib-0045] Chua JS , Liew HP , Guo L , Lane DP . Tumor‐specific signaling to p53 is mimicked by MDM2 inactivation in Zebrafish: insights from MDM2 and MDM4 mutant zebrafish. Oncogene. 2015;34:5933–41. 25746004 10.1038/onc.2015.57 PMC4661431

[pro4684-bib-0013] Coffill CR , Lee AP , Siau JW , Chee SM , Joseph TL , Tan YS , et al. The p53‐MDM2 interaction and the E3 ligase activity of MDM2/MDM4 are conserved from lampreys to humans. Genes Dev. 2016;30:281–92. 26798135 10.1101/gad.274118.115 PMC4743058

[pro4684-bib-0015] Dehal P , Boore JL . Two rounds of whole genome duplication in the ancestral vertebrate. PLoS Biol. 2005;3:e314. 16128622 10.1371/journal.pbio.0030314 PMC1197285

[pro4684-bib-0026] Eick GN , Bridgham JT , Anderson DP , Harms MJ , Thornton JW . Robustness of reconstructed ancestral protein functions to statistical uncertainty. Mol Biol Evol. 2016;34:msw223–61. 10.1093/molbev/msw223 PMC6095102 27795231

[pro4684-bib-0069] Evans R , O'Neill M , Pritzel A , Antropova N , Senior A , Green T , et al. Protein complex prediction with AlphaFold‐Multimer. *bioRxiv* . 2021 10.1101/2021.10.04.463034

[pro4684-bib-0052] Ferreon JC , Lee CW , Arai M , Martinez‐Yamout MA , Dyson HJ , Wright PE . Cooperative regulation of p53 by modulation of ternary complex formation with CBP/p300 and HDM2. Proc Natl Acad Sci U S A. 2009;106:6591–6. 19357310 10.1073/pnas.0811023106 PMC2672497

[pro4684-bib-0072] Fersht A . Structure and mechanism in protein science: a guide to enzyme catalysis and protein folding. New York: Macmillan; 1999.

[pro4684-bib-0066] Fraser HB . Modularity and evolutionary constraint on proteins. Nat Genet. 2005;37:351–2. 15750592 10.1038/ng1530

[pro4684-bib-0035] Fuxreiter M , Simon I , Friedrich P , Tompa P . Preformed structural elements feature in partner recognition by intrinsically unstructured proteins. J Mol Biol. 2004;338:1015–26. 15111064 10.1016/j.jmb.2004.03.017

[pro4684-bib-0041] Gianni S , Freiberger MI , Jemth P , Ferreiro DU , Wolynes PG , Fuxreiter M . Fuzziness and frustration in the energy landscape of protein folding, function, and assembly. Acc Chem Res. 2021;54:1251–9. 33550810 10.1021/acs.accounts.0c00813 PMC8023570

[pro4684-bib-0065] Han J‐DJ , Bertin N , Hao T , Goldberg DS , Berriz GF , Zhang LV , et al. Evidence for dynamically organized modularity in the yeast protein–protein interaction network. Nature. 2004;430:88–93. 15190252 10.1038/nature02555

[pro4684-bib-0031] Harms MJ , Thornton JW . Evolutionary biochemistry: revealing the historical and physical causes of protein properties. Nat Rev Genet. 2013;14:559–71. 23864121 10.1038/nrg3540 PMC4418793

[pro4684-bib-0057] He F , Borcherds W , Song T , Wei X , Das M , Chen L , et al. Interaction between p53 N terminus and core domain regulates specific and nonspecific DNA binding. Proc Natl Acad Sci U S A. 2019;116:8859–68. 30988205 10.1073/pnas.1903077116 PMC6500136

[pro4684-bib-0037] Hultqvist G , Åberg E , Camilloni C , Sundell GN , Andersson E , Dogan J , et al. Emergence and evolution of an interaction between intrinsically disordered proteins. elife. 2017;6:e16059. 28398197 10.7554/eLife.16059 PMC5419745

[pro4684-bib-0009] Ingaramo MC , Sánchez JA , Dekanty A . Regulation and function of p53: a perspective from drosophila studies. Mech Dev. 2018;154:82–90. 29800619 10.1016/j.mod.2018.05.007

[pro4684-bib-0040] Jemth P , Karlsson E , Vögeli B , Guzovsky B , Andersson E , Hultqvist G , et al. Structure and dynamics conspire in the evolution of affinity between intrinsically disordered proteins. Sci Adv. 2018;4:eaau4130. 30397651 10.1126/sciadv.aau4130 PMC6200366

[pro4684-bib-0002] Joerger AC , Fersht AR . The p53 pathway: origins, inactivation in cancer, and emerging therapeutic approaches. Annu Rev Biochem. 2016;85:375–404. 27145840 10.1146/annurev-biochem-060815-014710

[pro4684-bib-0004] Joerger AC , Wilcken R , Andreeva A . Tracing the evolution of the p53 tetramerization domain. Struct Lond Engl 1993. 2014;22:1301–10. 10.1016/j.str.2014.07.010 PMC4155161 25185827

[pro4684-bib-0068] Jumper J , Evans R , Pritzel A , Green T , Figurnov M , Ronneberger O , et al. Highly accurate protein structure prediction with AlphaFold. Nature. 2021;596:583–9. 34265844 10.1038/s41586-021-03819-2 PMC8371605

[pro4684-bib-0047] Karlsson E , Schnatwinkel J , Paissoni C , Andersson E , Herrmann C , Camilloni C , et al. Disordered regions flanking the binding interface modulate affinity between CBP and NCOA. J Mol Biol. 2022;434:167643. 35605677 10.1016/j.jmb.2022.167643

[pro4684-bib-0056] Krois AS , Dyson HJ , Wright PE . Long‐range regulation of p53 DNA binding by its intrinsically disordered N‐terminal transactivation domain. Proc Natl Acad Sci U S A. 2018;115:E11302–10. 30420502 10.1073/pnas.1814051115 PMC6275486

[pro4684-bib-0020] Kuraku S , Meyer A , Kuratani S . Timing of genome duplications relative to the origin of the vertebrates: did cyclostomes diverge before or after? Mol Biol Evol. 2008;26:47–59. 18842688 10.1093/molbev/msn222

[pro4684-bib-0018] Kussie PH , Gorina S , Marechal V , Elenbaas B , Moreau J , Levine AJ , et al. Structure of the MDM2 oncoprotein bound to the p53 tumor suppressor transactivation domain. Science. 1996;274:948–53. 8875929 10.1126/science.274.5289.948

[pro4684-bib-0003] Lane DP , Cheok CF , Brown C , Madhumalar A , Ghadessy FJ , Verma C . MDM2 and p53 are highly conserved from placozoans to man. Cell Cycle. 2010;9:540–7. 20081368 10.4161/cc.9.3.10516

[pro4684-bib-0010] Lane DP , Verma C . Mdm2 in evolution. Genes Cancer. 2012;3:320–4. 23150765 10.1177/1947601912458285 PMC3494368

[pro4684-bib-0044] Langheinrich U , Hennen E , Stott G , Vacun G . Zebrafish as a model organism for the identification and characterization of drugs and genes affecting p53 signaling. Curr Biol. 2002;12:2023–8. 12477391 10.1016/s0960-9822(02)01319-2

[pro4684-bib-0039] Laursen L , Čalyševa J , Gibson TJ , Jemth P . Divergent evolution of a protein‐protein interaction revealed through ancestral sequence reconstruction and resurrection. Mol Biol Evol. 2021;38:152–67. 32750125 10.1093/molbev/msaa198 PMC7782867

[pro4684-bib-0043] Lee K‐C , Goh WLP , Xu M , Kua N , Lunny D , Wong JS , et al. Detection of the p53 response in zebrafish embryos using new monoclonal antibodies. Oncogene. 2008;27:629–40. 17684488 10.1038/sj.onc.1210695

[pro4684-bib-0060] Levine AJ . p53: 800 million years of evolution and 40 years of discovery. Nat Rev Cancer. 2020;20:471–80. 32404993 10.1038/s41568-020-0262-1

[pro4684-bib-0028] Levrero M , De Laurenzi V , Costanzo A , Gong J , Wang JY , Melino G . The p53/p63/p73 family of transcription factors: overlapping and distinct functions. J Cell Sci. 2000;113(Pt 10):1661–70. 10769197 10.1242/jcs.113.10.1661

[pro4684-bib-0064] Már M , Nitsenko K , Heidarsson PO . Multifunctional intrinsically disordered regions in transcription factors. Chemistry. 2023;29:e202203369. 36648282 10.1002/chem.202203369

[pro4684-bib-0070] Malatesta F . The study of bimolecular reactions under non‐pseudo‐first order conditions. Biophys Chem. 2005;116:251–6. 15896898 10.1016/j.bpc.2005.04.006

[pro4684-bib-0019] McLysaght A , Hokamp K , Wolfe KH . Extensive genomic duplication during early chordate evolution. Nat Genet. 2002;31:200–4. 12032567 10.1038/ng884

[pro4684-bib-0038] Mihalic F , Simonetti L , Giudice G , Sander MR , Lindqvist R , Akprioro Peters MB , et al. Large‐scale phage‐based screening reveals extensive pan‐viral mimicry of host short linear motifs. Nat Commun. 2023;14:38015. 10.1038/s41467-023-38015-5 PMC10132805 37100772

[pro4684-bib-0025] Mirdita M , Schütze K , Moriwaki Y , Heo L , Ovchinnikov S , Steinegger M . ColabFold: making protein folding accessible to all. Nat Methods. 2022;19:679–82. 35637307 10.1038/s41592-022-01488-1 PMC9184281

[pro4684-bib-0012] Muttray AF , O'Toole TF , Morrill W , Van Beneden RJ , Baldwin SA . An invertebrate MDM homolog interacts with p53 and is differentially expressed together with p53 and ras in neoplastic *Mytilus trossulus* haemocytes. Comp Biochem Physiol B Biochem Mol Biol. 2010;156:298–308. 20417299 10.1016/j.cbpb.2010.04.008 PMC2916963

[pro4684-bib-0071] Nikolovska‐Coleska Z , Wang R , Fang X , Pan H , Tomita Y , Li P , et al. Development and optimization of a binding assay for the XIAP BIR3 domain using fluorescence polarization. Anal Biochem. 2004;332:261–73. 15325294 10.1016/j.ab.2004.05.055

[pro4684-bib-0016] Putnam NH , Butts T , Ferrier DEK , Furlong RF , Hellsten U , Kawashima T , et al. The amphioxus genome and the evolution of the chordate karyotype. Nature. 2008;453:1064–71. 18563158 10.1038/nature06967

[pro4684-bib-0029] Rozenberg JM , Rogovaya OS , Melino G , Barlev NA , Kagansky A . Distinct p63 and p73 protein interactions predict specific functions in mRNA splicing and polyploidy control in epithelia. Cell. 2020;10:25. 10.3390/cells10010025 PMC7824480 33375680

[pro4684-bib-0030] Rozenberg JM , Zvereva S , Dalina A , Blatov I , Zubarev I , Luppov D , et al. The p53 family member p73 in the regulation of cell stress response. Biol Direct. 2021;16:23. 34749806 10.1186/s13062-021-00307-5 PMC8577020

[pro4684-bib-0055] Sakaguchi K , Saito S , Higashimoto Y , Roy S , Anderson CW , Appella E . Damage‐mediated phosphorylation of human p53 threonine 18 through a cascade mediated by a casein 1‐like kinase. Effect on MDM2 binding. J Biol Chem. 2000;275:9278–83. 10734067 10.1074/jbc.275.13.9278

[pro4684-bib-0062] Sanborn AL , Yeh BT , Feigerle JT , Hao CV , Townshend RJ , Lieberman Aiden E , et al. Simple biochemical features underlie transcriptional activation domain diversity and dynamic, fuzzy binding to mediator. elife. 2021;10:e68068. 33904398 10.7554/eLife.68068 PMC8137143

[pro4684-bib-0023] Schon O , Friedler A , Bycroft M , Freund SMV , Fersht AR . Molecular mechanism of the interaction between MDM2 and p53. J Mol Biol. 2002;323:491–501. 12381304 10.1016/s0022-2836(02)00852-5

[pro4684-bib-0054] Sekelsky JJ , Brodsky MH , Burtis KC . DNA repair in *Drosophila*: insights from the drosophila genome sequence. J Cell Biol. 2000;150:F31–6. 10908583 10.1083/jcb.150.2.f31 PMC2180214

[pro4684-bib-0048] Shimizu H , Burch LR , Smith AJ , Dornan D , Wallace M , Ball KL , et al. The conformationally flexible S9–S10 linker region in the core domain of p53 contains a novel MDM2 binding site whose mutation increases ubiquitination of p53 in vivo. J Biol Chem. 2002;277:28446–58. 11925449 10.1074/jbc.M202296200

[pro4684-bib-0042] Shin J‐S , Ha J‐H , Lee D‐H , Ryu K‐S , Bae K‐H , Park BC , et al. Structural convergence of unstructured p53 family transactivation domains in MDM2 recognition. Cell Cycle. 2015;14:533–43. 25591003 10.1080/15384101.2014.998056 PMC4614862

[pro4684-bib-0024] Showalter SA , Bruschweiler‐Li L , Johnson E , Zhang F , Brüschweiler R . Quantitative lid dynamics of MDM2 reveals differential ligand binding modes of the p53‐binding cleft. J Am Chem Soc. 2008;130:6472–8. 18435534 10.1021/ja800201j

[pro4684-bib-0011] Siau JW , Coffill CR , Zhang WV , Tan YS , Hundt J , Lane D , et al. Functional characterization of p53 pathway components in the ancient metazoan *Trichoplax adhaerens* . Sci Rep. 2016;6:33972. 27678309 10.1038/srep33972 PMC5039725

[pro4684-bib-0032] Siddiq MA , Hochberg GK , Thornton JW . Evolution of protein specificity: insights from ancestral protein reconstruction. Curr Opin Struct Biol. 2017;47:113–22. 28841430 10.1016/j.sbi.2017.07.003 PMC6141201

[pro4684-bib-0017] Smith JJ , Kuraku S , Holt C , Sauka‐Spengler T , Jiang N , Campbell MS , et al. Sequencing of the sea lamprey (Petromyzon marinus) genome provides insights into vertebrate evolution. Nat Genet. 2013;45:415–21. 23435085 10.1038/ng.2568 PMC3709584

[pro4684-bib-0063] Staller MV , Ramirez E , Kotha SR , Holehouse AS , Pappu RV , Cohen BA . Directed mutational scanning reveals a balance between acidic and hydrophobic residues in strong human activation domains. Cell Syst. 2022;13:334–345.e5. 35120642 10.1016/j.cels.2022.01.002 PMC9241528

[pro4684-bib-0033] Steube N , Moldenhauer M , Weiland P , Saman D , Kilb A , Ramírez Rojas AA , et al. Fortuitously compatible protein surfaces primed allosteric control in cyanobacterial photoprotection. Nat Ecol Evol. 2023;7:756–67. 37012377 10.1038/s41559-023-02018-8 PMC10172135

[pro4684-bib-0058] Sun X , Dyson HJ , Wright PE . A phosphorylation‐dependent switch in the disordered p53 transactivation domain regulates DNA binding. Proc Natl Acad Sci U S A. 2021;118:e2021456118. 33443163 10.1073/pnas.2021456118 PMC7817127

[pro4684-bib-0008] Tan BX , Liew HP , Chua JS , Ghadessy FJ , Tan YS , Lane DP , et al. Anatomy of MDM2 and MDM4 in evolution. J Mol Cell Biol. 2017;9:3–15. 28077607 10.1093/jmcb/mjx002 PMC6372010

[pro4684-bib-0051] Teufel DP , Bycroft M , Fersht AR . Regulation by phosphorylation of the relative affinities of the N‐terminal transactivation domains of p53 for p300 domains and MDM2. Oncogene. 2009;28:2112–8. 19363523 10.1038/onc.2009.71 PMC2685776

[pro4684-bib-0050] Teufel DP , Freund SM , Bycroft M , Fersht AR . Four domains of p300 each bind tightly to a sequence spanning both transactivation subdomains of p53. Proc Natl Acad Sci U S A. 2007;104:7009–14. 17438265 10.1073/pnas.0702010104 PMC1855428

[pro4684-bib-0036] Tompa P , Davey NE , Gibson TJ , Babu MM . A million peptide motifs for the molecular biologist. Mol Cell. 2014;55:161–9. 25038412 10.1016/j.molcel.2014.05.032

[pro4684-bib-0014] Vandepoele K , De Vos W , Taylor JS , Meyer A , Van de Peer Y . Major events in the genome evolution of vertebrates: Paranome age and size differ considerably between ray‐finned fishes and land vertebrates. Proc Natl Acad Sci. 2004;101:1638–43. 14757817 10.1073/pnas.0307968100 PMC341801

[pro4684-bib-0001] Vogelstein B , Lane D , Levine AJ . Surfing the p53 network. Nature. 2000;408:307–10. 11099028 10.1038/35042675

[pro4684-bib-0049] Wallace M , Worrall E , Pettersson S , Hupp TR , Ball KL . Dual‐site regulation of MDM2 E3‐ubiquitin ligase activity. Mol Cell. 2006;23:251–63. 16857591 10.1016/j.molcel.2006.05.029

[pro4684-bib-0034] Wright PE , Dyson HJ . Intrinsically disordered proteins in cellular signalling and regulation. Nat Rev Mol Cell Biol. 2015;16:18–29. 25531225 10.1038/nrm3920 PMC4405151

[pro4684-bib-0061] Zarin T , Tsai CN , Nguyen Ba AN , Moses AM . Selection maintains signaling function of a highly diverged intrinsically disordered region. Proc Natl Acad Sci. 2017;114:E1450–9. 10.1073/pnas.1614787114 28167781 PMC5338452

[pro4684-bib-0027] Zdzalik M , Pustelny K , Kedracka‐Krok S , Huben K , Pecak A , Wladyka B , et al. Interaction of regulators MDM2 and MDMx with transcription factors p53, p63 and p73. Cell Cycle. 2010;9:4584–91. 21088494 10.4161/cc.9.22.13871

[pro4684-bib-0005] Zhang Q , Balourdas D‐I , Baron B , Senitzki A , Haran TE , Wiman KG , et al. Evolutionary history of the p53 family DNA‐binding domain: insights from an *Alvinella pompejana* homolog. Cell Death Dis. 2022;13:214. 35256607 10.1038/s41419-022-04653-8 PMC8901663

