# Supplementary material for: Evolution of affinity between p53 transactivation domain and MDM2 across the animal kingdom demonstrates high plasticity of motif‐mediated interactions
Source: Protein Sci. 2023 Jul 1;32(7):e4684. doi: 10.1002/pro.4684 (PMC10303687; doi:10.1002/pro.4684)
Supplement: Supplementary file 1 — Appendix S1: Supporting Information [file PRO-32-e4684-s006.pdf]

Supplementary Information

Evolution of affinity between p53 transactivation domain and MDM2 across the animal kingdom demonstrates high plasticity of motif-mediated interactions

Filip Mihalic[1,2], Emma Åberg[1,2], Pouria Farkhondehkish[1], Niels Theys[1], Eva Andersson[1], and Per Jemth[1]*

[1]Department of Medical Biochemistry and Microbiology, Uppsala University, BMC Box 582, SE-75123 Uppsala, Sweden.

[2]Equal contribution

*Corresponding author: Per Jemth, e-mail: per.jemth@imbim.uu.se

Supplementary Figures

Supplementary Fig. S1. Sequence alignment of the p53 transactivation domain and MDM2 SWIB domain.

Supplementary Figure S2. ITC experiments with p53TAD and MDM2 from different animals.

Supplementary Figure S3. Thermodynamic stability of MDM2 variants.

Supplementary Figure S4. ColabFold predictions of p53TAD/MDM2 complexes.

Supplementary Figure S5. Colabfold prediction for the *M. trossulus* complex compared to the crystal structure model of the human complex.

Supplementary Figure S6. Sequence alignment of p53TAD from tetrapods.

Supplementary Figure S7. Sequence alignments of TADs from p53, p63 and p73 from vertebrates.

Supplementary Tables, see separate files

Supplementary Excel File 1. Amino acid sequences of proteins and peptides used in the experiments.

Supplementary Excel File 2. Calculation of K_D values from FP experiments.

Supplementary Excel File 3. Reconstructed MDM2 sequences.

Supplementary Excel File 4. Reconstructed p53-family TAD sequences.

Supplementary text files, see separate files

Supplementary Text File 1. Alignment of TADs from extant p53, p63 and p73 proteins.

Supplementary Text File 2. Full-length sequences of extant p53, p63 and p73 proteins.

Supplementary Text File 3. Alignment of extant MDM2 proteins.

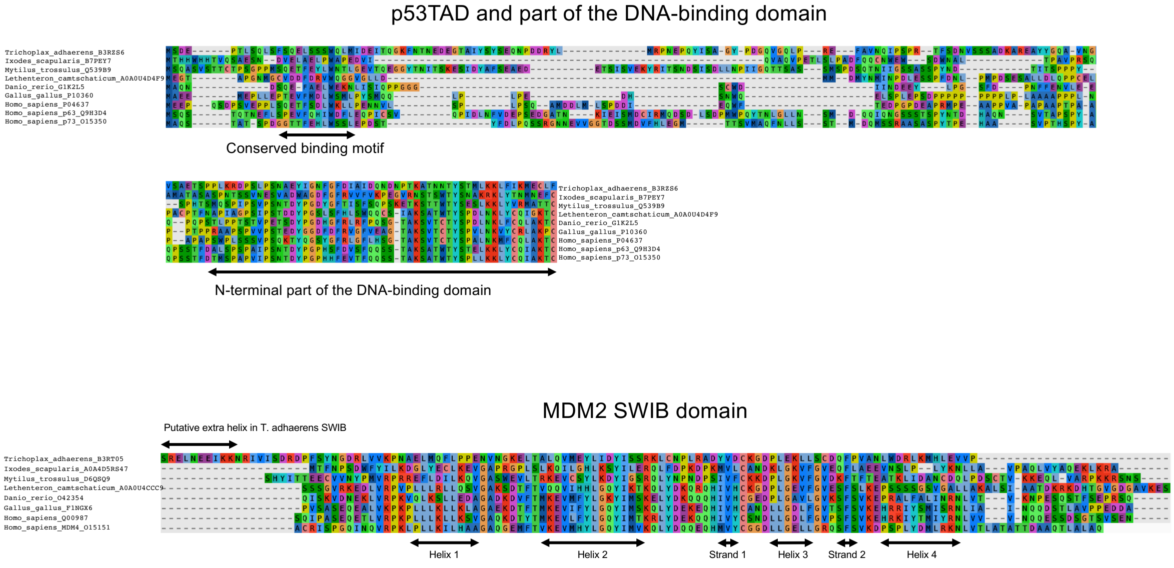

Figure S1

Supplementary Fig. S1. Sequence alignment of the p53 transactivation domain and MDM2 SWIB domain. (A) Sequence alignment of p53TADs from different species included in the study as well as the human paralogs p63 and p73. The sequence from the N-terminus to the beginning of the DNA-binding domain is shown for each protein. The alignment shows that the sequences have been subject to extensive evolution as reflected in the difference in amino acid sequences in proteins from extant animals. The large number of insertions and deletions precludes a correct alignment outside of the conserved binding motif. *Lethenteron camtschaticum* contains two additional p53 family proteins (Uniprot A0A0U4B546 and A0A0U3KDC1). The phylogeny of the three *L. camtschaticum* p53 paralogs in relation to gnathostome p53, p63 and p73 is not clear. Furthermore, the binding motifs appear to be lost in two of the paralogs and we have here aligned the sequence of the third one, with a remaining putative MDM2-binding motif. **(B)** Sequence alignment of the p53TAD-binding SWIB domains of MDM2 included in the study and of human MDM4. The sequence alignments were visualized with eBioX.

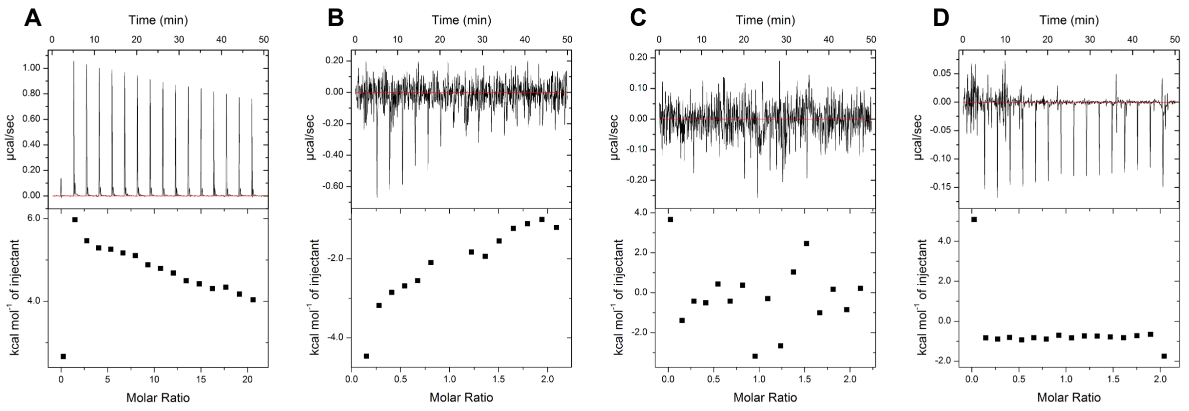

Supplementary Figure S2. ITC experiments with p53TAD and MDM2 from different animals. Binding could not be detected between several p53TAD/MDM2 pairs. **(A)** p53TAD$_{\textit{T.adhaerens}}$ was titrated into MDM2 $_{\textit{T.adhaerens}}$, **(B)** p53TAD$_{\textit{M.trossulus}}$ was titrated into MDM2$_{\textit{M.trossulus}}$, **(C)** p53TAD$_{\textit{I.scapularis}}$ was titrated into MDM2$_{\textit{I.scapularis}}$ and **(D)** p53TAD$_{\textit{L.camtschaticum}}$ was titrated into MDM2 $_{\textit{L.camtschaticum}}$.

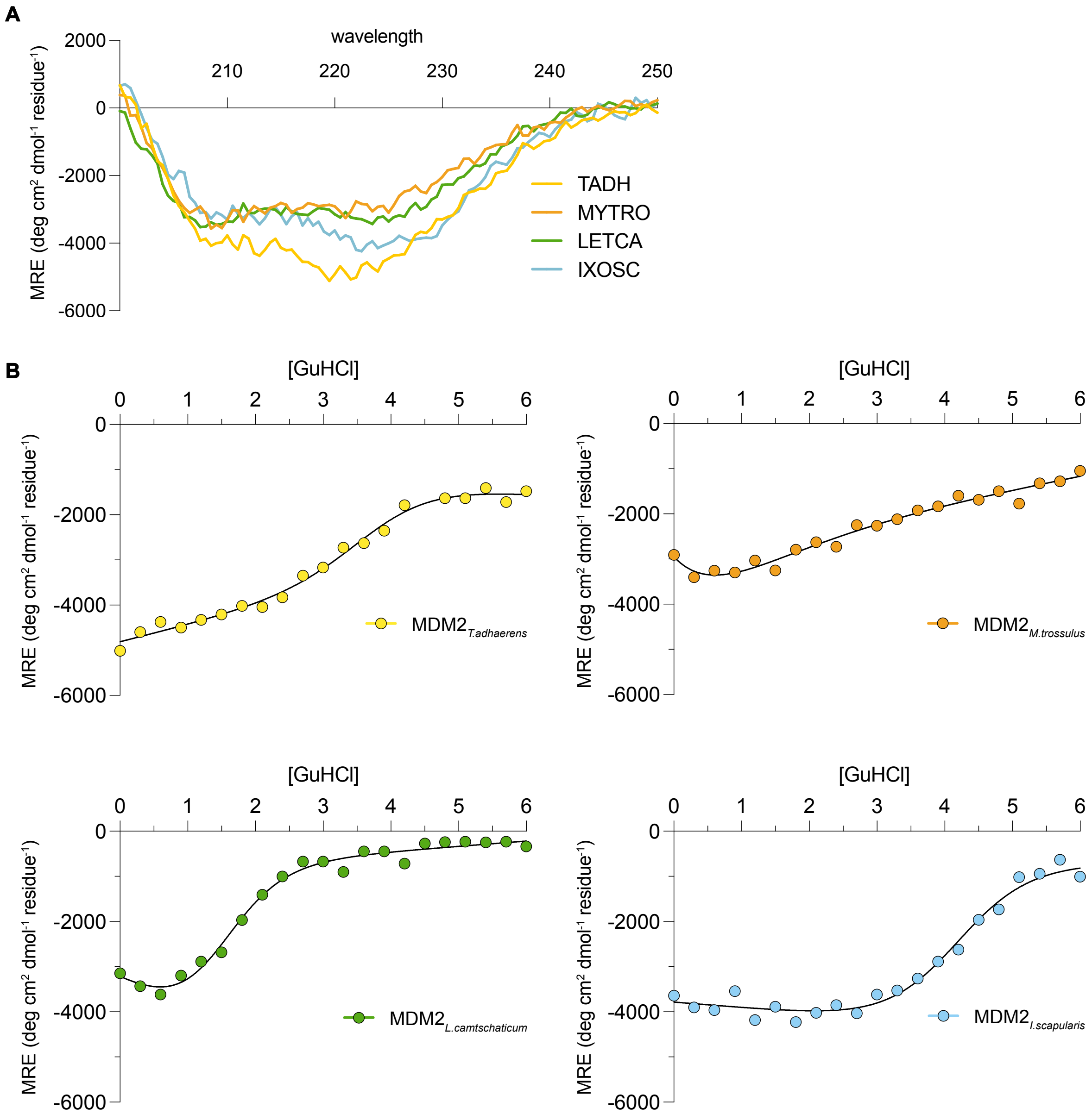

Supplementary Figure S3. Thermodynamic stability of MDM2 variants. (A) Circular dichroism spectra of native and denatured MDM2 SWIB domain from *T. adhaerens*, *M. trossulus, L. camtschaticum* and *I. scapularis* respectively. **(B)** Guanidinium chloride-mediated denaturation of the domains monitored at 222 nm suggest that the proteins are folded. Solid lines correspond to fits to a two-state model. MRE, molar ellipticity.

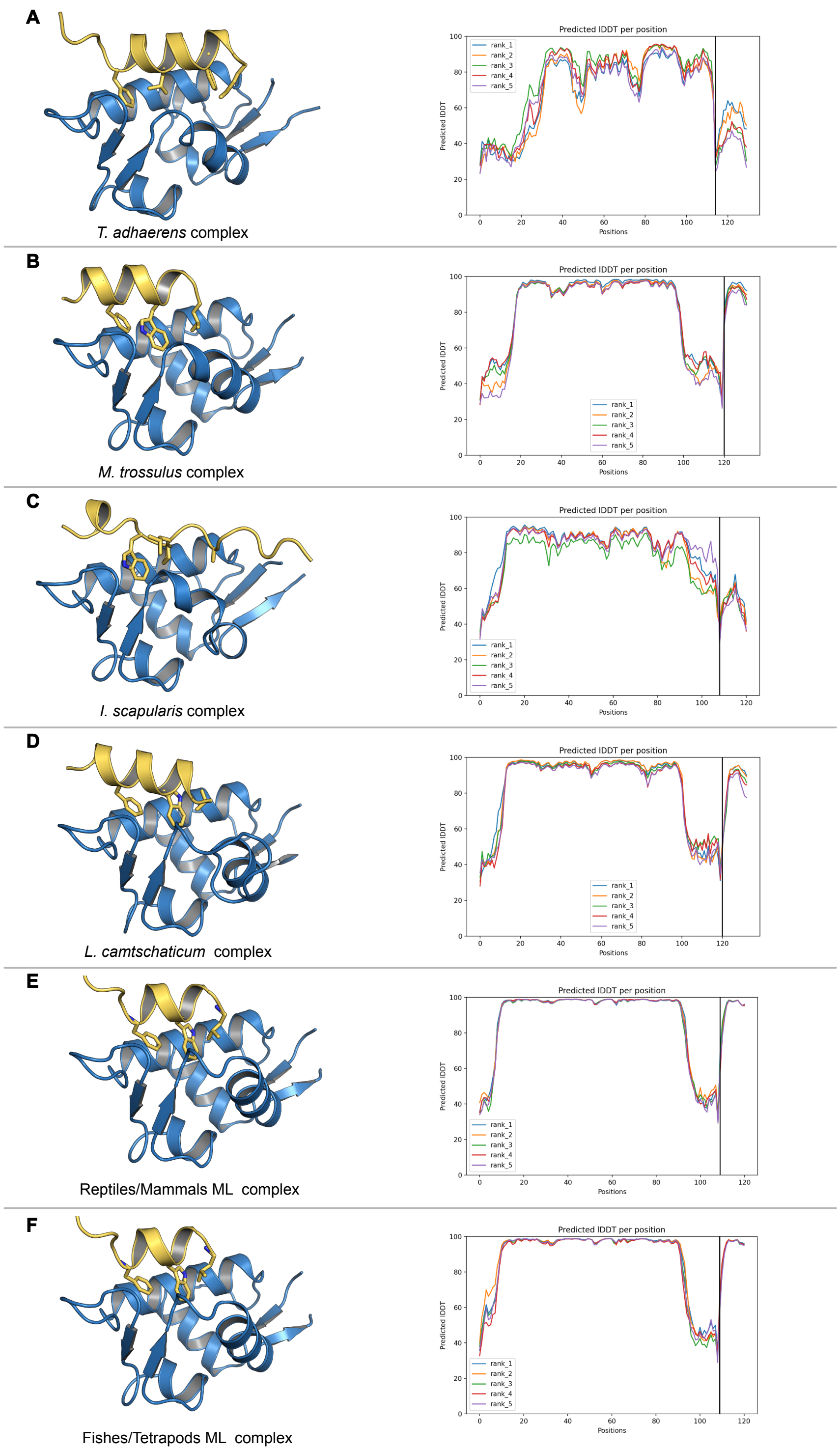

Supplementary Figure S4. ColabFold predictions of p53TAD/MDM2 complexes. p53TAD is in yellow and MDM2 in blue. **(A)** Prediction for *T. adhaerens* complex with binding of the extended motif (FxxxLxxxWxxM). **(B)** Prediction for *M. trossulus* complex. **(C)** Prediction for *I. scapularis* complex. **(D)** Prediction for *L. camtschaticum* complex. **(E)** Prediction for the resurrected ancestral reptiles/mammals complex. **(F)** Prediction for the resurrected ancestral fishes/tetrapods complex. In all cases, the p53TAD residues Phe19, Trp23 and Leu26 pointing into the hydrophobic pocket are shown as sticks.

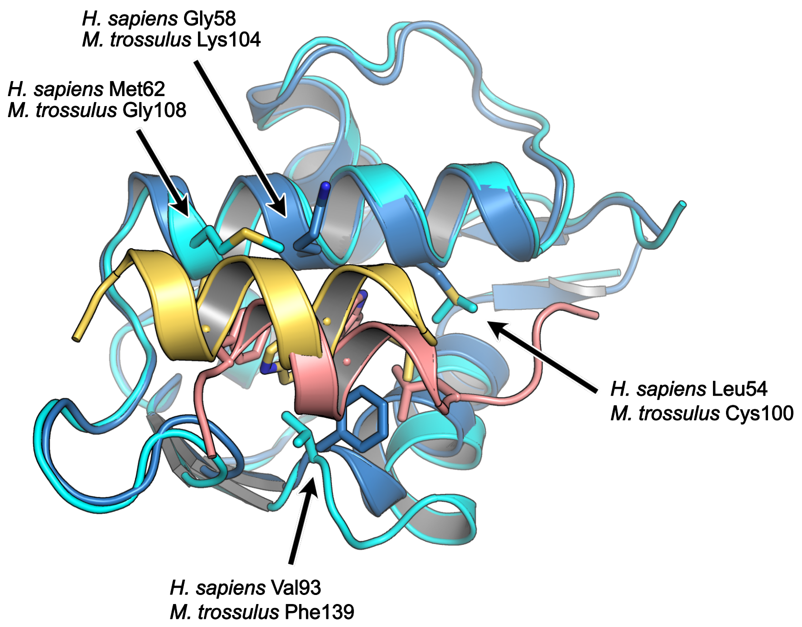

Supplementary Figure S5. Colabfold prediction for the *M. trossulus* complex compared to the crystal structure model of the human complex. The human MDM2 is in cyan (PDB id: 1YCR) [7], human p53TAD in salmon, *M. trossulus* MDM2 in blue and *M. trossulus* p53TAD in yellow. Arrows indicate key differences between human and *M. trossulus* MDM2 that likely provide a hydrophobic p53TAD binding pocket with sub-optimal p53TAD binding properties (amino acid numbering according to human MDM2 protein).

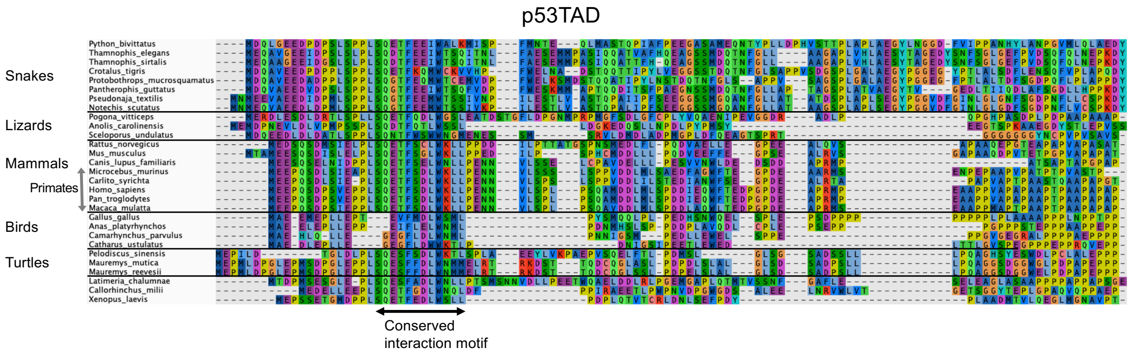

Supplementary Figure S6. Sequence alignment of p53TAD from tetrapods. Alignment of p53TAD colored according to sequence similarity. p53TAD can only be confidently aligned for closely related species such as primates. The alignment was performed using Muscle and adjusted manually in the N-terminus for birds and *P. sinensis*. Note that both phosphorylation sites Ser15 and Thr18 have been lost in the bird p53s. The figure was made in AliView.

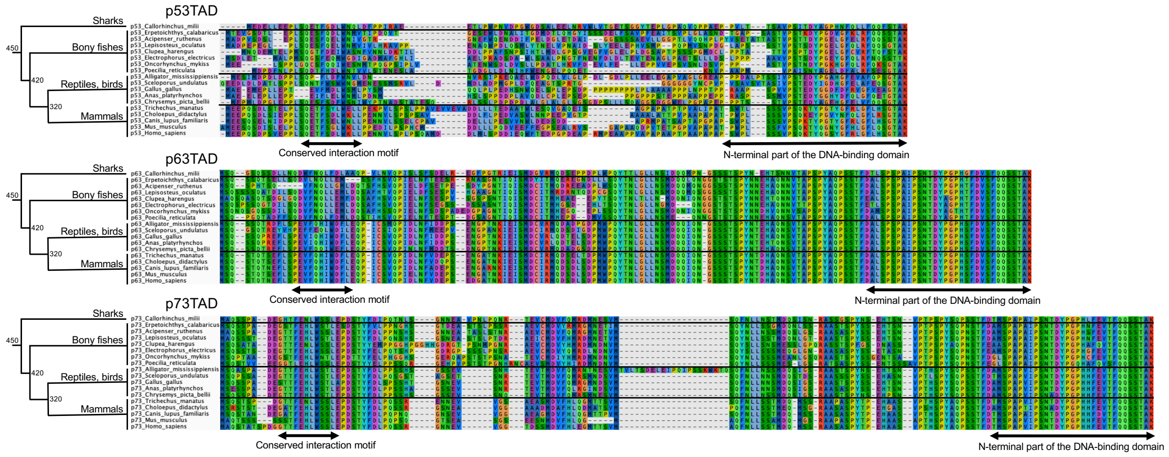

Supplementary Figure S7. Sequence alignments of TADs from p53, p63 and p73 from vertebrates. It is clear that the TADs of p63 and p73 are more conserved than that from p53. For p63 and p73, the alignment starts at the Met residue closest to the canonical binding motif. Thus, annotations in Uniprot or NCBI may include additional residues in the N-terminus. The long insertion in *Alligator mississippiensis* p73TAD (TVLTSDELEIPQIPSSKWKTQ) is present in its crocodilian relative *Crocodylus porosus* but not in *Gavialis gangeticus* p73TAD. The alignment was performed using Muscle and the figure was made in AliView. The numbers at the nodes of the phylogenetic tree shows the approximate time of divergence in million years ago.

Supplementary Excel File 1. Sequences of constructs used in the experiments. The sequence and name for each interaction motif in p53TAD and MDM2 SWIB domain used in binding experiments in the study.

Supplementary Excel File 2. Calculation of K_i from IC50 values. IC50 values were determined as described in the materials section. The theory behind the conversion of IC50 values to K_i values (=K_D) is described in Nikolovska-Coleska, *et al.*, Development and optimization of a binding assay for the XIAP BIR3 domain using fluorescence polarization. *Anal. Biochem.* **332**, 261–273 (2004).

Supplementary Excel File 3. Reconstruction of the SWIB domain of MDM2. Reconstructed maximum likelihood (ML) and low probability AltAll versions of the MDM2 SWIB domain. The posterior probability is shown for each amino acid for each position for the reconstructed sequences.

Supplementary Excel File 4. Reconstruction of p53TAD. Reconstructed maximum likelihood (ML) and low probability AltAll versions of the conserved interaction motif in p53TAD. The posterior probability is shown for each amino acid for each position for the reconstructed sequences.

Supplementary Text File 1. Fasta file for p53TAD. The sequence alignment file used for ancestral reconstruction of the interaction motif in p53TAD.

Supplementary Text File 2. Full length sequences of p53. Full length sequences of p53s used in the reconstruction. This file contains sequences that were removed in the final reconstruction, such as echinoderms, hemichordates and agnatha (lampreys).

Supplementary Text File 3. Fasta file for MDM2 SWIB. The sequence alignment file used for ancestral reconstruction of the SWIB domain of MDM2.
